# Unraveling mysteries of personal performance style; biomechanics of left-hand position changes (shifting) in violin performance

**DOI:** 10.7717/peerj.1299

**Published:** 2015-10-01

**Authors:** Peter Visentin, Shiming Li, Guillaume Tardif, Gongbing Shan

**Affiliations:** 1 Department of Music, University of Lethbridge, Lethbridge, Alberta, Canada; 2 College of Physical Education, Ludong University, Yantai, Shandong, China; 3 Department of Music, University of Alberta, Edmonton, Alberta, Canada; 4 Department of Kinesiology, University of Lethbridge, Lethbridge, Alberta, Canada

**Keywords:** 3D motion capture, Biomechanical modeling, Fine and complex human motor control, Anthropometry, Entrainable, Personal artistic style

## Abstract

Instrumental music performance ranks among the most complex of learned human behaviors, requiring development of highly nuanced powers of sensory and neural discrimination, intricate motor skills, and adaptive abilities in a temporal activity. Teaching, learning and performing on the violin generally occur within musico-cultural parameters most often transmitted through aural traditions that include both verbal instruction and performance modeling. In most parts of the world, violin is taught in a manner virtually indistinguishable from that used 200 years ago. The current study uses methods from movement science to examine the “how” and “what” of left-hand position changes (shifting), a movement skill essential during violin performance. In doing so, it begins a discussion of artistic individualization in terms of anthropometry, the performer-instrument interface, and the strategic use of motor behaviors. Results based on 540 shifting samples, a case series of 6 professional-level violinists, showed that some elements of the skill were individualized in surprising ways while others were explainable by anthropometry, ergonomics and entrainment. Remarkably, results demonstrated each violinist to have developed an individualized pacing for shifts, a feature that should influence timing effects and prove foundational to aesthetic outcomes during performance. Such results underpin the potential for scientific methodologies to unravel mysteries of performance that are associated with a performer’s personal artistic style.

## Introduction

Instrumental music performance ranks among the most complex of learned human behaviors. It requires intricate motor skills, perception and adaptation in a temporal endeavor, and sensory and neural discrimination that challenges the limits of human cognition (Doidge, 2007; Huron, 2007; Levetin, 2007; Sachs, 2008; Snyder, 2000; Taylor, 2010). From a phenomenological point of view, music performance shares many characteristics with other skill-oriented activities (Chesky et al., 2002; Lehmann & Davidson, 2002; Wilson, 1986). In particular, commonalities between artistic and athletic activities are obvious in their dependence on motor skill development—both acquisition during learning and execution during performance. However, the use of technology and scientific method in teaching and learning music performance has lagged far behind its use in sports. Even today, classical western music pedagogy continues to perpetuate its traditions mainly through a one-on-one apprenticeship learning model. In such a model, conventions and aesthetic values are transmitted from teacher to pupil via aural tradition and, as part of the process, the learner must practice many hours of repetitive exercises to perfect complex motor control sequences. In most parts of the world, classical musicians today are taught in a manner virtually indistinguishable from that used 200 years ago. Nowhere in the world could the same be said for elite sport.

Given successes that have been achieved by applying scientific methods in athletic training, it seems logical to adapt these to the context of music performance. In a 2002 comprehensive review, Kennell acknowledged “growing professional interest in applying the tools of systematic research to the context of studio instruction in music education research” (Kennell, 2002). None of the studies cited addressed any aspect of teaching the biomechanical skills requisite for successful musical performance (Flohr & Hodges, 2002). Since then, a few studies have begun to quantitatively examine motor behavior performance elements for bowed stringed instruments (representative articles cited) (Deliège & Wiggins, 2006; Hargreaves, Miell & MacDonald, 2012; Papadelis, 2006; Shan & Visentin, 2003; Shan et al., 2007; Visentin, Shan & Wasiak, 2008). None of these deal with the topic of the current paper, a tempo-dependent analysis of left-hand position changes in violin performance. Further, although all of these articles describe phenomena associated with motor behavior during motor activities needed for playing an instrument, none do so in a manner that begins to consider a context close to that of a performance.

The dearth of such work may partly be explained by the fact that, in great part, success in music performance is typically determined by how an audience responds to an artist’s playing. This necessarily involves the artist’s manipulation of many different factors to create an effective end-result. Defining effectiveness in this context involves negotiating the slippery slope of musico-cultural expectations. Compounding complexity is the fact that there are few “absolutes” in the process. For example, even the most basic element of music, tuning of pitch, is internally referential to the performance since the western scale of 12 chromatic pitches has a built-in imperfection (for more discussion of the Pythagorean tuning system, see Appendix A). As such, tuning becomes as much a matter of tone, execution, dynamic nuance and musical context as it is of pitch frequency. As well, repeatability is not generally an artist’s goal. Rather, modification of selected elements during performance is normally invoked as a response to past events or as part of a forward planning process. Hence, the manner in which accomplishment is both achieved and defined can be vague. Such realities pose challenges for controlling of variables in scientific studies, making qualitative assessment a desirable adjunct to quantitative measurements in the study of music performance.

Finally, when adapting methods from Sports Science to a music performance context, it must be understood the focus of training differs substantively between the two. In athletics, much of training concentrates on understanding motor control qualities governing strength and balance (gross motor control), while in many music endeavors it involves nuanced movements associated with biofeedback responses (fine motor control). Even the words used to describe the process of skill acquisition show differential cultural loading; the words “training” and “practicing” are used in sport, whereas all music learning sessions are conceived of as “practice” for musicians. The absence of the word “training” in the musician’s consciousness suggests there to be a greater concern with the end product than with the process of achieving it.

Scientific methods can be used to quantify elements of artistic practice. In instrumental music performance movement analysis can help demystify skill acquisition processes by addressing the “how” and “what” of motor control. However, can such studies begin to unravel mysteries of performance that are sometimes associated with musicians’ motivational “why”: the development of personal artistry? Movement science is highly relevant to performers since it can help accelerate skill acquisition processes and improve effectiveness during performance (Visentin, Shan & Wasiak, 2008). Efficiency improvements in skill execution help reduce the amount of time that a performer must spend practicing. Previous studies have determined that repetitive movements associated with long hours of practice and performance are a main cause of the development of musculoskeletal injuries (MSI) among musicians (Brandfonbrener, 2003; Dawson, 2002; Ranelli, Straker & Smith, 2008; Zaza, 1998). Among professional violinists in orchestras, injury rates are more than 75% (Dawson, 1990; Fishbein et al., 1988; Zaza, 1998). Many of these injuries are lifelong; some are career-ending. Notwithstanding this well-documented reality, performing artists primarily concern themselves with the end product. Practices that are perceived as negating individuality or limiting artistic possibilities are considered to be antithetical to the creation of good art (Shan & Visentin, 2010). For musicians, concepts of effectiveness and individualization are inseparable from skill acquisition. Hence, research intended to reach music practitioners must address all of these elements.

One aim of the current study is to better understand the skill of left-hand position changes (shifting) in terms that might make it easier for violinists to acquire and automate in the context of learning and performance. A second aim is to initiate discussion on matters hitherto relegated to the mysticism of the artistic process—relationships among those elements which are measurable and generalizable and those that may be particular to an artist. In performance, these work together. The current study contributes to a body of research that can: (1) improve efficiency of violinists’ skill acquisition; (2) increase effectiveness in their motor behaviors (which can free them to focus on outcomes that express their creative muse); and (3) ultimately lead to the prevention of musculoskeletal injuries among violinists by providing fundamental knowledge necessary for the development of pedagogical best practices. A multidisciplinary approach to research provides the means to accomplish these goals. The current study uses methods from movement science to identify biomechanical mechanisms of a select skill vital for violin performance—namely shifting—and discusses them in the context of motor behaviors developed through lengthy practice, anthropometry and quality of skill execution.

### Background

Motor control during shifting is extremely complex. To better understand the role that shifting plays in violin performance, a general overview of the mechanics of playing the violin is necessary. The violin has four strings. The four fingers of the left hand are used to press the strings onto a fingerboard, changing the string lengths to give pitches of varied frequencies. The thumb is used as an opposable digit and does not participate directly in pitch production. The placement of the left hand relative to the distal end of the fingerboard determines the pitch that each finger is able to play and each discrete placement is identified as a left hand “position.” A numbering system is used to distinguish one position from another (e.g., 1st position, 2nd position, etc.—distal to proximal). Shifting is the act of moving from one position to another; moving from a lower numbered position to a higher one is called shifting “up” and the reverse is identified as shifting “down.” Because of the physical properties of acoustics, pitches are logarithmically distributed along each given string, making even basic motor control an exercise in non-linear spatial memory (Kazennikov & Wiesendanger, 2009). Shifting, moving from one position to another, compounds the complexity of finger placement because a performer’s hand starts in one non-linear special orientation and ends in another. The most common control strategy for performers is to use a “guide” finger (Dounis, 1921), normally the finger played just before the shift, which remains in contact with the string (1) creating a subtle but audible sonic reference during the shift, and (2) intensifying somatosensory bio-feedback via multiple finger and hand contact points with the instrument, both of which aid the triangulation of distances and positioning of the hand.

**Figure 1:**
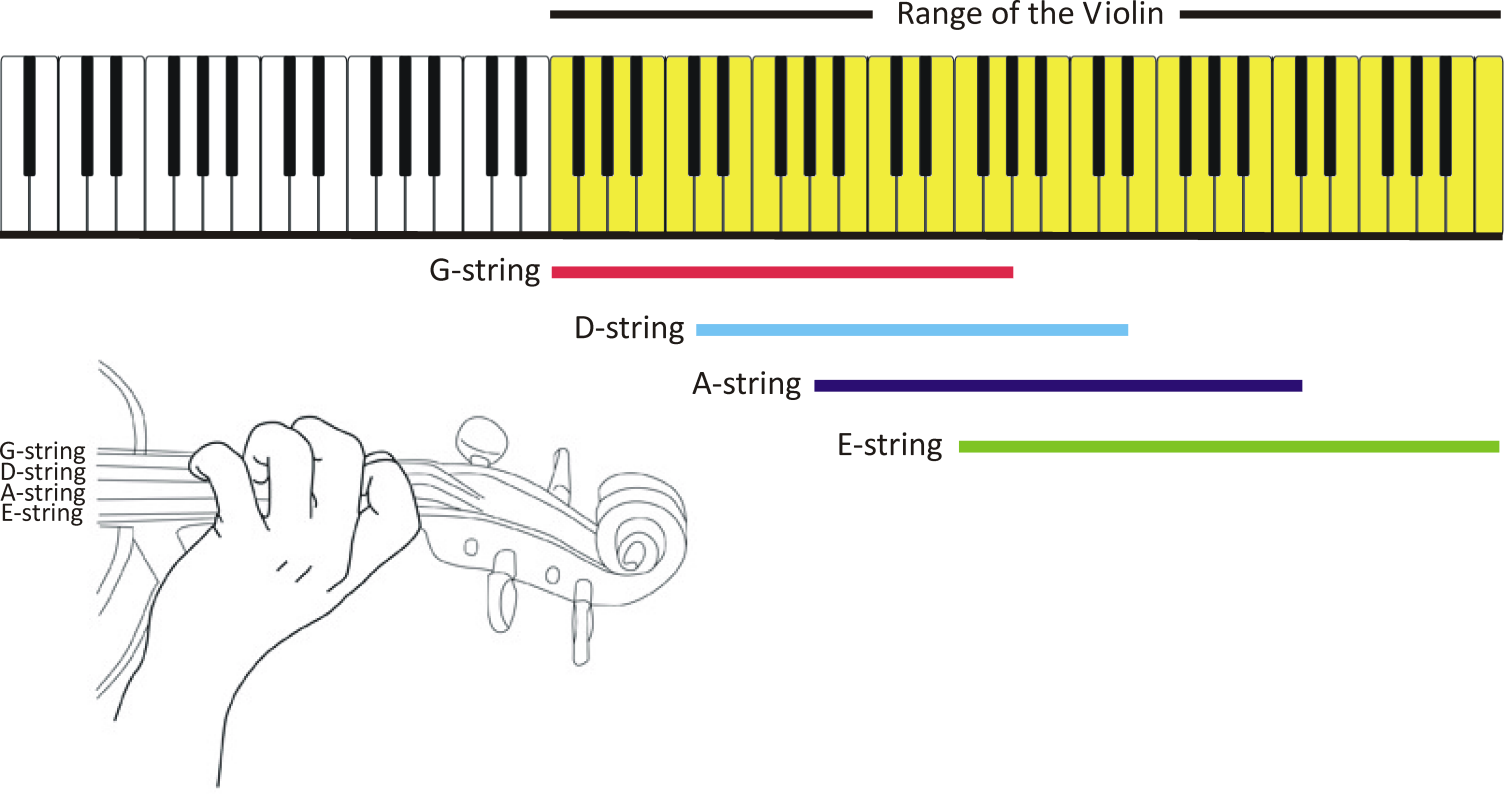
Pitch possibilities of the piano and the violin compared (figure created by the authors).

Without shifting, only 29 different pitches of the western music scale are possible on the violin. With shifting, not only are more pitches available but some notes can be played on multiple strings, resulting in more than 100 possible pitches of varied acoustic-spectral signatures or timbres. Thus, in artful performance shifting may be used as an expressive tool, and not merely as a utilitarian means of generating pitch frequencies. As a matter of comparison, the piano has 55 note possibilities in the same pitch range as the violin (Fig. 1).

Although a large number of books and trade publications have been written about violin technique, most are comprised of empirical observations that repeat the directives typically relayed to learners during lessons. Some sources merely provide practice exercises while others include more elaborate descriptors, representative examples cited (Dounis, 1921; Galamian, 1962; Gerle, 1983; Ševčík, 1905). Most are written in such a manner that only someone already familiar with the phenomenon of playing a string instrument can understand them. Hence, such documentation typically supplements the aural traditions and experienced-based learning methods prevalent in western music pedagogy. A search of the literature reveals very few quantitative scientific studies of violin technique (Baader, Kazennikov & Wiesendanger, 2005; Shan & Visentin, 2003; Visentin, Shan & Wasiak, 2008). No quantitative studies examine tempo-dependent (playing speed), left-hand motor control. The current study examines such control for the skill of shifting during the performance of a standard training composition from the violin literature.

## Materials & Methods

The test protocol was scrutinized and approved by the Human Subjects Research Committee of the University of Lethbridge as the protocol meets the criteria from the Tri-Council Policy Statement: Ethical Conduct for Research Involving Humans, from the Natural Sciences & Engineering Research Council. All subjects in the study were informed of the testing procedures. They signed an approved consent form and voluntarily participated in the data collection. Data collection was carried out in accordance with approved guidelines.

A 3-D motion-capture system was used to measure full-body movement using 68 reflective markers—39 on the body, 22 on the left hand, 4 on the violin and 3 on the bow. A twelve-camera VICON MX40 motion capture system (VICON Motion Systems, Oxford Metrics Ltd., Oxford, England) tracked the markers at a rate of 200 frames/s. Figure 3 shows a 3-D computer reconstruction the capture set-up. Use of 12 cameras and small markers permitted considerable freedom of movement for the subjects, ensuring subjects’ movements within the capture volume remained as close to their normal “style” as possible. Subjects were all of professional level, having between 18 and 46 years of experience studying and performing on the violin. Three were male and three were female. Raw kinematic data was processed using a five-point, weighted average (1-3-4-3-1 function) smoothing filter, which reduced the effects of noise from possible vibration of the markers during movement.

**Figure 2:**
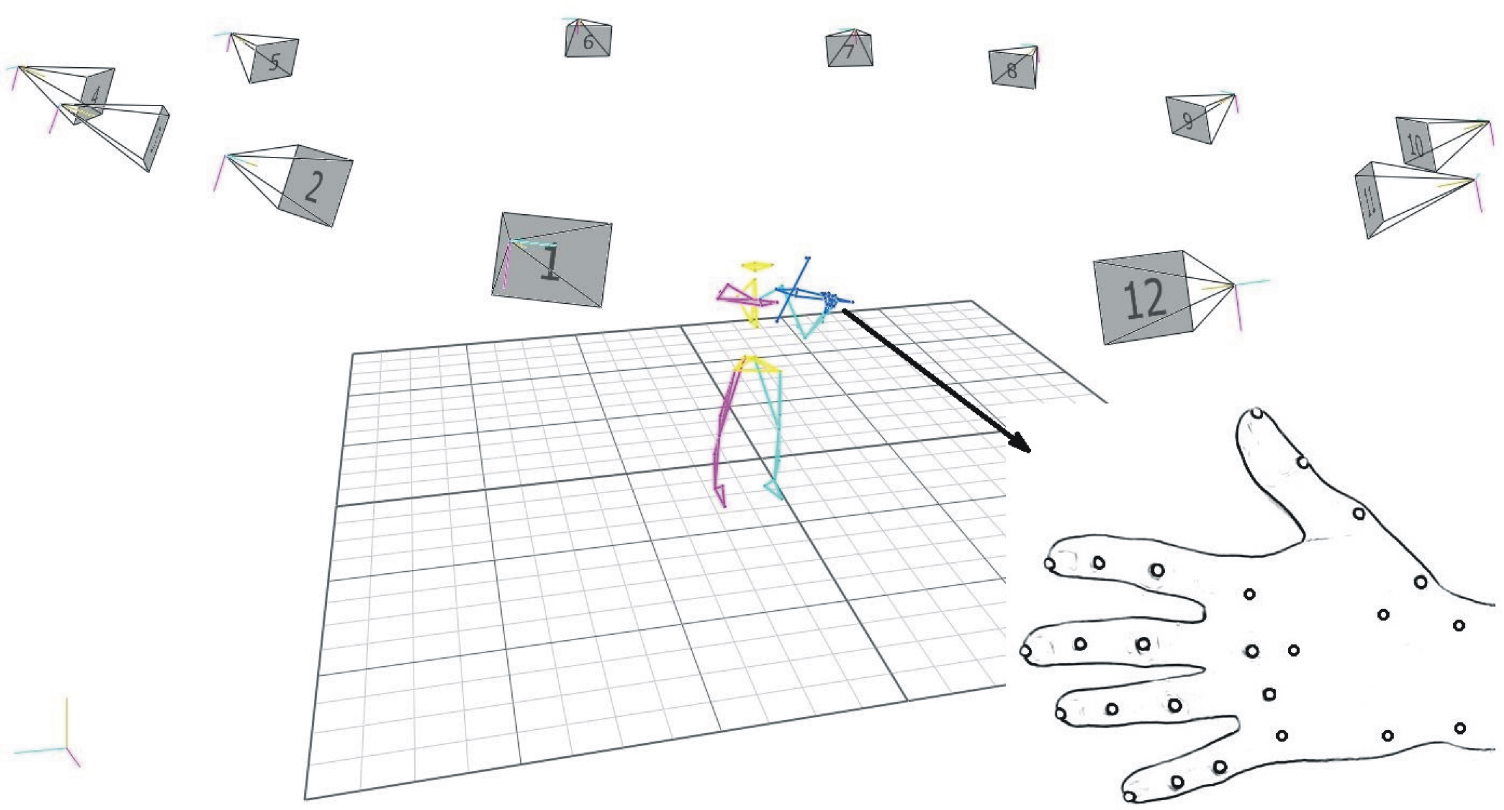
3-D motion capture set-up (12 high-speed cameras), subject reconstruction (biomechanical model), left-hand marker placement.

Thirty-nine body markers (9 mm in diameter) were used to build a 15-segment full-body biomechanical model (Shan et al., 2015; Shan & Westerhoff, 2005; Visentin et al., 2010; Zhang & Shan, 2014). Markers were placed on subjects as follows: on the head (4) sternal end of the clavicle, xiphoid process of the sternum, C7 and T10 vertebrae, right scapula, left and right anterior superior iliac, posterior superior iliac, right and left acromion, lateral side of each upper arm, lateral epicondyles, lateral side of forearms, styloid processes of radii and ulnae, distal ends of 3rd metacarpal bones, left and right lateral sides of thighs and shanks, lateral tibial condyles, lateral malleoli, calcanei and big toes. Twenty-two markers (3 mm semi-spherical) were placed on the left-hand (Fig. 2). Since the model design needed to document coordination of the left hand with the violin and the movement of the bow, additional markers were placed as follows: on the violin, one on the tailpiece and scroll, with two on the bridge end of the fingerboard; on the bow, three markers, one on the button and two on the head. Standard biomechanical frames of reference are used for flexion/extension, ab/adduction and rotation of joins and segments (Hall, 2006).

**Figure 3:**
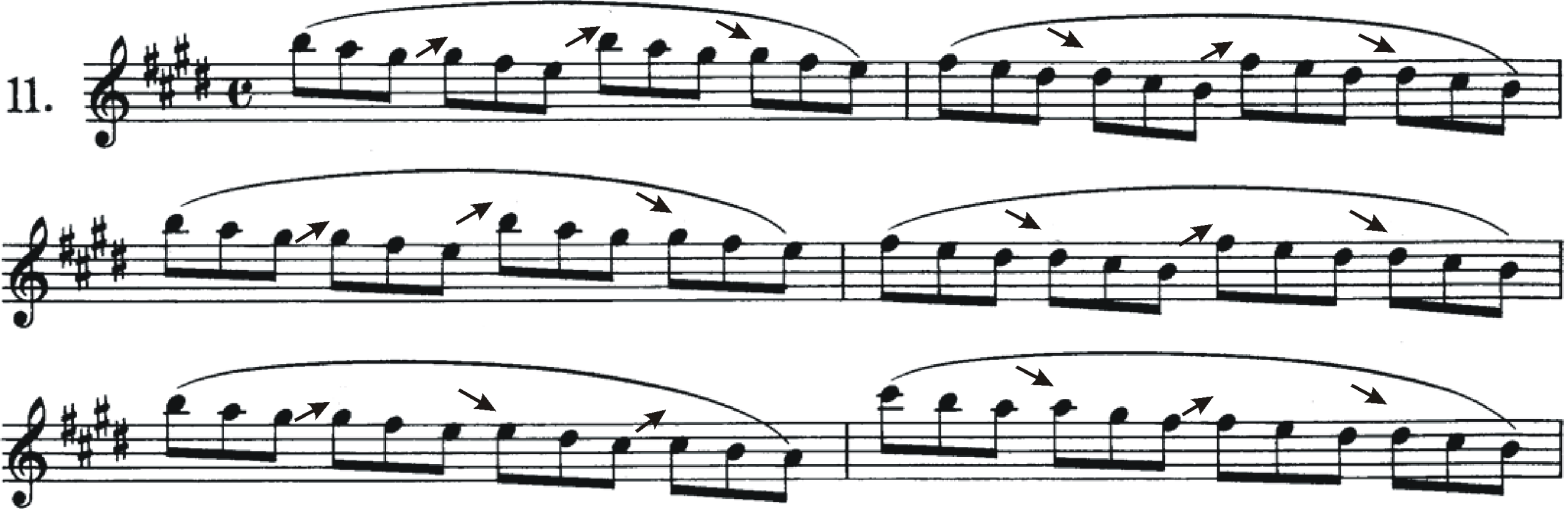
First six bars of etude #11 by Kreutzer (1796). Arrows are used to identify shift placements and their directionality (up or down).

An excerpt from the etudes of Kreutzer (1796) provided subjects with a familiar test composition (Fig. 3). This compendium occupies an almost unique position in the literature of violin studies in terms of the universality of its use in pedagogy, even today (Charlton, 2001). Etudes are exercises with musical elements selectively exploited to develop a player’s skills. Written in 1796, the 11th etude focusses on shifting as a training exercise in a metrical, harmonically organized context typical of the musical literature from the common practice historical eras (Baroque Classic and to great extent Romantic eras). The chordal organization makes a prominent feature of intonation (tuning sensitivity) and, combined with frequent position changes, the composition demands players find optimized movement processes to maintain a steady pace.

Eighteen shifts (9 up, 9 down) were identified in the first six bars of the composition. These were categorized into 9 motor control patterns based on: (1) starting and ending points of the shift, (2) fingers used, and (3) the string(s) played during the shift (Table 1). All of these shifts move two positions (the pitch interval of a 3rd), the most common kind employed in violin playing. Shifting patterns I, VII and IX begin on one string and end on another, requiring string crossings as part of both the left hand and the bowing arm motor control mechanisms. As a matter of note, since the fingerboard projects over top of the body of the violin, positions higher than the 4th typically require the violinist to accommodate for the curvature of the violin body, usually accomplished by pronating the left arm through shoulder abduction and rotation. Joint angles and ranges of motion (ROM) of left-arm joint angles are examined to document this effect.

**Table 1:** Shifting patterns in the first six bars of Kreutzer Etude #11.

Shifting patterns	Shift #	Finger starting the shift	Finger ending the shift	Start position	End position	String(s) used
I	1,7,13	middle	small	1st	3rd	E, A
II	2,8	middle	small	3rd	5th	A
III	3,9	middle	small	5th	3rd	A
IV	4,6,10,12,18	index	ring	3rd	1st	A
V	5,11	index	ring	1st	3rd	A
VI	14	middle	small	3rd	1st	A
VII	15	middle	small	1st	3rd	A, D
VIII	16	index	ring	3rd	1st	E
IX	17	index	ring	1st	3rd	E, A

**Notes.**

For violin, fingers are numbered in music as follows: 1, 2, 3, 4 = index, middle, ring, small, respectively.

The use of a metronome (a device that generates a steady beat) during trials provided a timing reference for subjects to coordinate the precision of onsets (the articulatory beginning of notes) from beat to beat. Duration of shifting (DOS) was determined using frame by frame visual identification of finger, hand and elbow markers in the reconstructed three-dimensional motion capture data from each trial. The end of each shift was established by the sudden stop (sudden deceleration—increase in |Δ*a*|) of a finger-tip as it arrived in contact with the violin fingerboard, beginning a new pitch. Since shifting involves the entire arm of the player, the initiation of each shift was defined as the moment of first continuously directed movement of an anatomic landmark involved in the shifting process (one of the thumb, wrist, or elbow markers). The DOS was calculated by subtracting initiation from ending times. Timing accuracy of shift endings (the time differentials between consecutive shift endings) was found by determining the number of motion capture frames between these events and dividing it by the 200 frame-per-second capture rate. Trials were repeated at each of three different speeds: two trials each at 60 and 72 beats per minute (b/min) and one trial at 100 b/min. Since the composition required performers to play three notes per beat of the metronome, participants played three notes per second at the slowest tempo (60 b/min) and five at the fastest (100 b/min). Using this protocol, 90 shifts per participant were measured resulting in a database of 540 shifts in total. Descriptive statistics (averages, standard deviation, ANOVA) were derived using SPSS. Multiple comparisons (Scheffe) were used to examine intra-individual significances related to tempo.

Since shifting provides a means to an end in violin performance, definitions of the success of motor behaviors associated with the skill should at least partly consider aural outcomes. To accomplish this in the current study, a questionnaire provided a tool for qualitative evaluation of the trials. Three professional musicians, (all with more than 25 years of professional work experience) evaluated recordings of the shifting trials for each of the six subjects of this study (the first trial at each performance speed). Aural evaluation is the standard examination tool in the music industry. By definition it is qualitative. In practice, it is the only kind of evaluation that is accepted by musicians. In orchestral auditions, prospective orchestra members audition for a job by performing from behind a screen so that their performance is evaluated only on the aural result. Some orchestras have gone so far as to disallow foot-ware at auditions, lest the sounds of the shoes allow identification of applicants’ genders. Adjudicator’s evaluations are grounded in experience—oft times based on thousands of performances and listening experiences from their past. Four questions were asked for each trial: (1) evaluate the overall execution of the excerpt; (2) evaluate the overall execution of the shifts; (3) evaluate the shifting intonation; and (4) evaluate the timing and steadiness of the playing. Responses were graded on a five-point Likert scale (1 = poor, fair, good, very good, excellent = 5). The average of all scores for each subject across all three tempi and for all three adjudicators provided a means to numerically compare the aural results of the performances.

## Results

At any tempo, the articulatory beginning (the onset) of every pitch must be rhythmically precise or listeners will hear an audible timing differential. One study has shown bimanual bow and finger coordination differentials of 70–100 ms to be tolerable in terms of musical feedback during violin playing (Kazennikov & Wiesendanger, 2009). The current study employed a test composition that required performers to play 12 notes and as many as 3 left hand shifts on each stroke of the bow. With few bow direction changes to distract attention from left-hand timing phenomena, sensitivity to both audible timing errors of the left-hand fingers and pitch accuracy is high. Further, because timing effects are cumulative, either a performer must be 100% accurate (an impossible task) or make subtle adjustments to remain in phase with the beat overall. Results of the current study revealed that, among the highly trained subjects of the current study, average end of shift timing (EST) accuracies ranged from 27 to 68 milliseconds (ms) (Table 2). Notwithstanding the fact that 9 different shifting patterns were employed, there is no relationship between shifting patterns and EST accuracies. Fifteen of the eighteen tests show average EST accuracies of 46 ms or less. Only two subjects, S4 and S6, show any significant tempo-dependent differences in EST accuracies, those occurring between the mid and fast tempi (60 & 100 b/min), *p* = 0.045 for each.

**Table 2:** Characteristics of shifting and anthropometry (bold: significantly different *p* < 0.05). Error values are 5 ms based on the motion capture frame rate (200 f/s).

Subjects		Body Height (m)	End of shift timing (ms)			Duration of shift (ms)		
			60 b/min	72 b/min	100 b/min	60 b/min	72 b/min	100 b/min
Male	S1	1.86	36 ± 33	41 ± 35	42 ± 33	317 ± 40	308 ± 38	300 ± 30
	S2	1.77	33 ± 23	29 ± 22	46 ± 44	404 ± 57	404 ± 62	389 ± 45
	S3	1.75	55 ± 67	36 ± 46	68 ± 49	**333 ± 40**	319 ± 39	**294 ± 33**
Female	S4	1.66	**27 ± 21**	40 ± 19	**46 ± 33**	364 ± 18	**376 ± 24**	**351 ± 42**
	S5	1.60	28 ± 21	43 ± 37	36 ± 27	302 ± 57	309 ± 34	305 ± 34
	S6	1.50	**32 ± 26**	35 ± 20	**52 ± 36**	461 ± 42	453 ± 43	455 ± 43

Average DOS (the total time from first initiation of the shift to the moment of sounding the arrival pitch) ranged from 294 to 461 ms. We began the study expecting that shifting would be highly related to tempo; remarkably, every subject seems to have acquired his/her own individualized speed for shifting. DOS timing is extremely stable for the subjects in this study (Table 2). With the exception of S3, average DOS timing across all tempi vary 25 ms or less. Table 2 shows two subjects to have had statistically significant tempo-dependent DOS variability. DOS became smaller as the playing speed became faster for S3, with a significant difference occurring between 60 and 100 b/min, *p* < 0.03. For S4, data indicates shifts to be slower for 72 b/m than for 60 b/m (*p* > 0.05) and significantly faster again between 72 and 100 b/min (*p* < 0.01).

**Figure 4:**
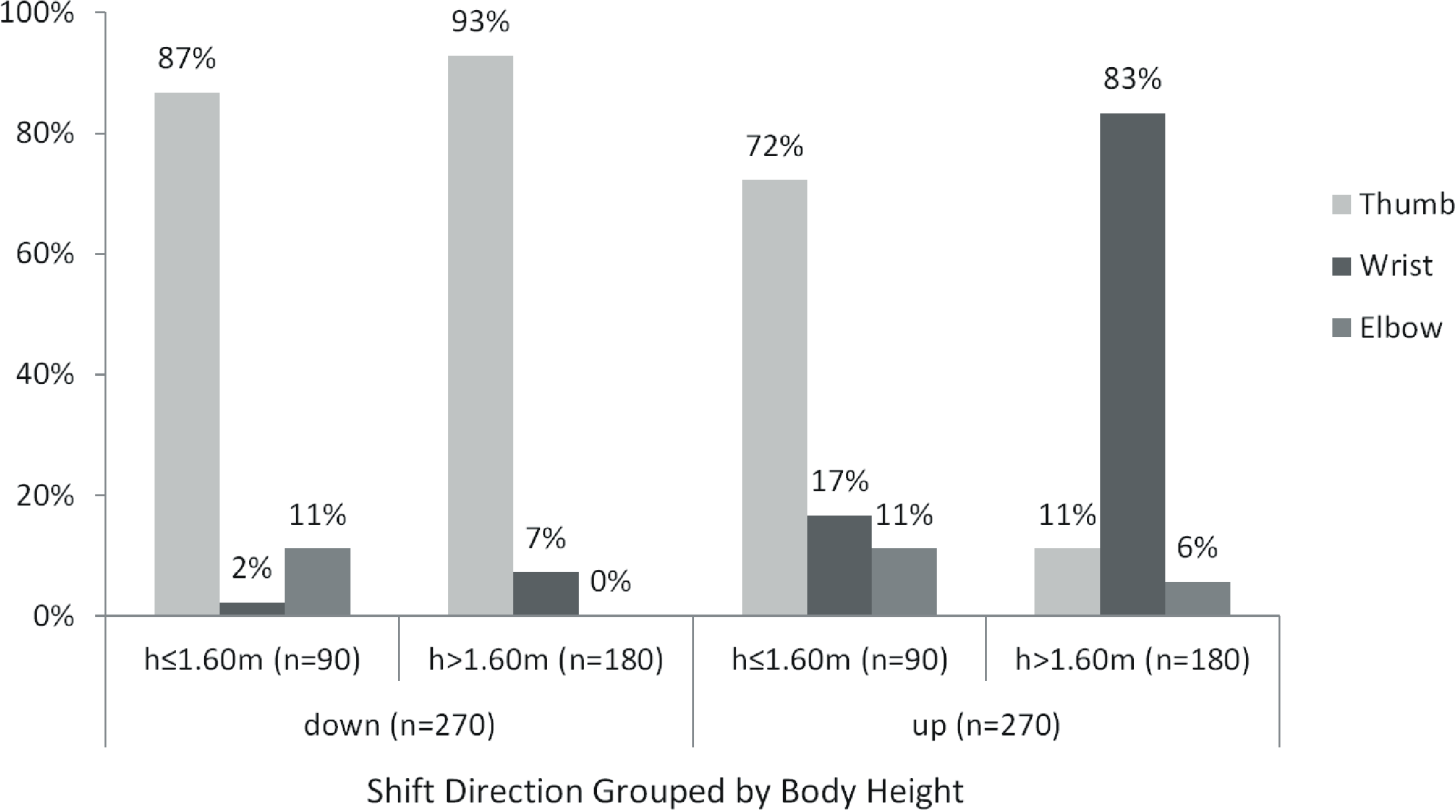
Initiator frequency related to shift direction and body height.

The initiation of each shift was invariably signaled by movement in markers associated with one of three left limb locations: thumb, wrist or elbow. Figure 4 shows motor control initiators grouped by shift direction and body height of the subjects. For shorter subjects (*h* ≤ 1.60 m), the thumb was the dominant initiator regardless of shift direction (87% for downward and 72% for upward shifts). Notably, taller subjects employed a changing strategy, 93% of downward shifts were initiated with the thumb and 83% of upward ones employed the wrist. Strategies based on level of training and individualized control characteristics can be observed in contexts where wrist and elbow initiators were employed only sparingly (≤17%). Only two individuals used a lateral movement of the elbow as an initiation strategy (Table 3). This movement was accomplished by shoulder ab/adduction and rotation. Each of these subjects was consistent in their behavior regarding elbow control. Notably, S6 was the only subject to employ a change of initiator strategy to accommodate tempo increases, and that only at the fastest tempo.

**Table 3:** Subjects employing thumb, wrist and elbow as shifting initiators.

	Height (m)	Initiator	Tempo			Total
			60	72	100	
S3 (male)	1.75	Thumb	39%	39%	39%	39%
		Wrist	39%	39%	39%	39%
		Elbow	22%	22%	22%	22%
S6 (female)	1.50	Thumb	89%	89%	44%	82%
		Wrist	0%	0%	44%	9%
		Elbow	11%	11%	11%	9%

Three-dimensional kinematic data revealed two general finger control strategies among subjects: (1) grouping, and (2) action-by-action. In a grouping technique, a “guide” finger remains on the string during shifting until the finger ending the shift has reached its new position, sometimes remaining on the string even after the new pitch has sounded. Five of the six subjects dependably employed this technique. The exceptional subject (S3), the only one to use finger control where the guide finger function was either truncated or absent, had significant tempo-dependence in DOS (Table 2, 60–100 b/min, *p* = 0.003) as well as the greatest variability and largest standard deviations in EST of all subjects (Table 2).

Table 4 shows the influence of anthropometry on the motor strategies of the shortest and tallest of the subjects. Angle maxima show the most acute postures reached by each joint during performance, while ROM shows the amount of movement found in the joint. Most of the kinematic data indicates remarkable differences (Table 4, highlighted cells). The most extreme differences in angle acuity were found in the shoulder rotation and flexion/extension. ROM shows that the main compensatory mechanisms for negotiating the ergonomics of the instrument are found in shoulder abduction and in all three components of the wrist. ROM of shoulder ab/adduction is more than twice as large for S6 as it is for S1 (Table 4). All components of wrist control show dramatically larger ROM for S6 compared to S1 (Table 4). Notably, ROM of wrist rotation for S6 is more than three times than that of S1.

**Table 4:** The influence of anthropometry on left-arm control strategies—ranges of motion (ROM) compared for tallest and shortest subjects.

		Subject 1		Subject 6	
		Max. (°)	ROM (°)	Max. (°)	ROM (°)
Shoulder	Flex/ext	8.9 ± 0.3	9.5 ± 0.3	16.2 ± 1.4	11.0 ± 0.8
	Abd/add	11.1 ± 0.5	6.8 ± 0.5	13.4 ± 1.0	15.1 ± 0.6
	Rotation	24.7 ± 0.5	13.6 ± 0.4	38.1 ± 1.4	12.7 ± 0.5
Elbow	Flex/ext	50.7 ± 0.5	10.7 ± 0.7	59.0 ± 0.7	13.1 ± 0.8
Wrist	Flex/ext	139.0 ± 0.7	25.9 ± 0.9	109.2 ± 1.7	37.6 ± 1.1
	Abd/add	39.3 ± 0.7	10.0 ± 0.8	38.7 ± 0.9	23.1 ± 0.9
	Rotation	63.4 ± 0.8	11.0 ± 0.5	73.8 ± 0.4	36.8 ± 1.3

**Figure 5:**
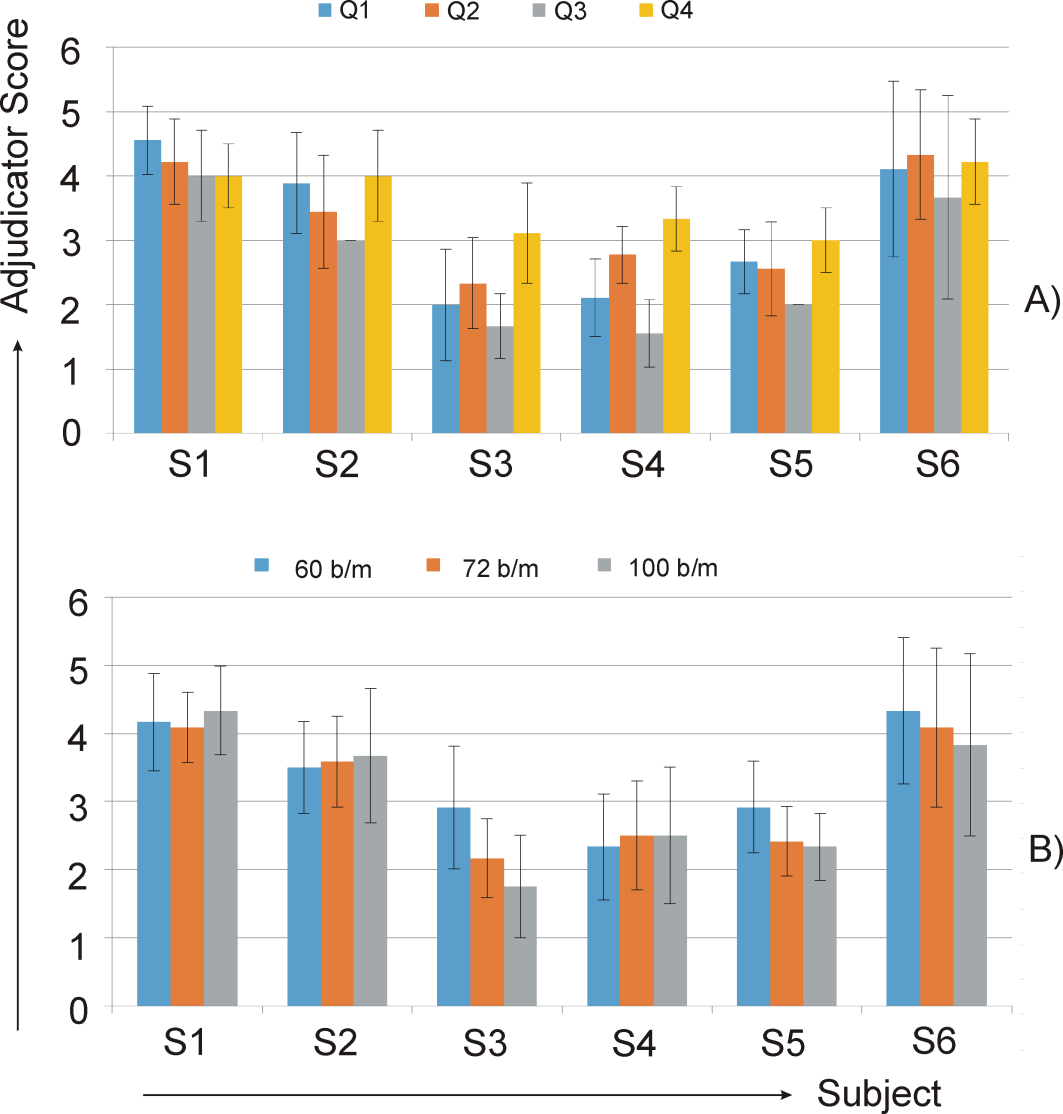
Questionnaire evaluation: (A) average of all adjudicators’ responses for each subject at each tempo; (B) average of all adjudicators’ responses to each survey question (all tempi) for each subject.

In terms of performance outcomes, qualitative assessments of the three music adjudicators, indicate S1 and S6 to have performed with a high degree of aural success (Fig. 5A). Results for S3 and S4 were least well-received. For S1, S2, and S4, average assessment scores indicate audible results to be nominally more successful at the highest tempo. The opposite holds for S3, S5 and S6—lower scores at higher tempi indicate less successful audible results, although this effect is only notably so for S3. An examination of survey results for each question reveals S1 and S6 to have consistently high results across all four questions (FIg. 5B). For the rest of the subjects, a general trend emerged that intonation accuracy (Q3) received the lowest scores and timing accuracy received the highest.

## Discussion

Since a violin is fixed in size, playing it requires a performer to find playing strategies adapted to his/her anthropometry. Statistical studies show that a 40 cm body height differential (the difference between S1 and S6 in the current study) normally results in 36% and 38% differences in hand and arm lengths respectively (Shan & Bohn, 2003). The scope of motor behavior possibilities for each performer may be substantially defined by such differences, but the strategic ways in which an individual operates within his/her own scope of possibilities become signature elements of his/her technique/artistry. These are developed over long periods of practice and performance.

We began this study expecting to find the duration of shifts (DOS) to be both influenced by tempo and vary due to the performance context. First, it seemed intuitively reasonable that, to go faster, one has to go faster. Thus, we expected DOS to become shorter at faster tempi. Second, complexity of shifts can vary which should influence timing (some of the shifts in the current study required synchronous changes of string and a concomitant height adjustment in the position of the right arm). Greater variability in shifting times based on context complexity was expected. With the exception of one subject (S3, Table 2), neither of these hypotheses was true for the tempi tested. With regard to DOS one subject (S4), actually lengthened DOS (slowed the shift down) from the slowest to the middle tempo. The reason for this is not clear from the quantitative data alone; however, given the data from survey question #3, we speculate that taking more time for shifts may have been a deliberately invoked strategy to stabilize intonation elements of the playing. It is notable that S3 and S4, the only subjects with statistically significant variance in DOS timing achieved the lowest scores in aural evaluations of recordings. The other performers were highly consistent in each their EST and DOS. This seems to suggest that, optimization of shifting tends to occur when timing elements of motor control are stabilized for skill execution; thus, the shifting process is resistant to tempo-based adaptation. This result adds to the findings of Repp (1994), where a key concept in studies of motor behavior—proportional duration—was tested for two performers playing Robert Schumann’s piano composition “Träumerei.” Repp found that proportional duration for main elements of expressive microstructure generally held across the tempi tested. Unlike Repp, the current study shows each violinist to have found his or her own pacing for shifts, an “inner rhythm” which might be related to training, anthropometry and ergonomics. The difference between these findings may suggest that expressive microstructures are as much a function of instrument played (the biomechanics of music performance) as they are of aural traditions in music.

The influence of anthropometry on shifting speed is clearly observable between the tallest and shortest of the subjects. S1, the tallest subject, had anthropometric advantage (e.g., longer fingers and arm segments) allowing him to cover shifting distances quickly with minimal flexion of the elbow, a joint involving gross motor control mechanisms. S6, the shortest subject, needed to employ more elbow flexion, resulting in the slowest shifts (longest durations) among subjects. DOS of S6 were substantially longer than for S1, something that might on the surface suggest that they were of lesser quality. From the standpoint of the adjudicator evaluations, this is clearly not the case. S1 and S6 are the most highly ranked subjects in all four questions of the survey, notwithstanding anthropometry or the differences in their motor behaviors. Neither of the motor control strategies identified can be considered superior to the other.

Anthropometry clearly limits some of the motor behavior choices of S6, the shortest of the subjects. For S6, four control characteristics were identifiable: (1) DOS was longest of any subject, (2) unlike any of the other subjects, S6 clearly preferred a thumb initiator for both shifting directions (Table 3), (3) for pattern number II (the highest position shifts of the excerpt) she initiated shifts with adduction and rotation of the shoulder as a means to anticipate working around the curvature of the violin, and (4) at the fastest speed, S6 changed from a thumb to a wrist dominant strategy for upward shifts. These trade-offs are, in great part, necessary because of her size. For S6, assessments of audio recordings reveal intonation to be highly accurate (Fig. 5, Q3). Notably, the shift from a thumb to a wrist initiation strategy for the fastest tempo did not affect her overall DOS timing or intonation, but it did negatively affect EST accuracy (*p* = 0.045) (Table 2). Although statistically significant, the size of this effect was only 17 ms. Thus, the overall success of her performance suggests that the strategies employed by S6 may be taken as an archetypal starting point for teaching very short violinists.

Further, anthropometry plays a clear role in negotiating the ergonomics of the violin. Comparing S1 (tallest) and S6 (shortest), ergonomic compensation can be observed in two different parameters: maximum joint angle and ROM (Table 4). Except for wrist abduction/adduction, all joints operate at larger angles for S6 than for S1 (due to anatomic angle definitions, a larger value in wrist flex/ext indicates less flexion). In this regard, shoulder rotation and wrist flex/ext are notable for the large differences observed. The maximum wrist rotation for S6 indicates her to be working close to, or at, anatomic limits. For ROM, shoulder ab/adduction and all components of the wrist are factors in the compensation process. For S6, larger abduction of the shoulder occurs and the forearm must be more highly pronated (as measured by wrist rotation), especially in the higher positions. All of the above may explain why DOS time for S6 is about 50% longer than that of S1 (Table 2).

Underlying principles in motor theory state that accuracy decreases as joints controlling larger segments become more active and moving larger segments takes more effort and influences stability of fine motor control (Magill, 2001). These principles appear to be born out in the data from initiator strategies, where individualization seems strongly related to anthropometry. S1 and S4, the tallest (and the most experienced) of each of the male and female subjects, were entirely consistent in their initiator strategy. Both used wrist flexion to initiate upward shifts. For upward shifting, by initiating with the wrist (moving the hand) rather than the elbow or shoulder (moving the whole arm), these players minimize ranges of motion in joints responsible for gross motor control, adding stability to the action. Shifting downward for these two subjects involved the coordination of three segments during the shift; the thumb moved first to release contact pressure from the neck of the violin, wrist and elbow extension followed to cover distance. Exceptionally, in the highest positions S1 (the tallest of the subjects) exclusively employed extension of the wrist for downward shifts. In these positions, he was able to cover the distances required while having the heel of the hand remain in contact with the curved body of violin as a stabilizing influence. It appears that for downward shifts in higher positions S1 developed a playing strategy that married his anthropometric potential with the ergonomics of the instrument. Strategies of S2 and S5 may be explained as finding a middle ground between S4 and S6, both of whom used combinations of thumb and wrist initiators in consistent patterns. For S6, her small stature necessitated greater use of the elbow and shoulder (in higher positions) than for any other subject, which may account for the longer length of her DOS. On the other hand, S3 employed shoulder adduction and rotation (laterally moving the elbow) similar to S6, notwithstanding that, given his height, there seems to be no anthropometric driver for this choice. His hand and arm were sufficiently large to use strategies similar to those employed by S2 or S4. This choice of motor control, one that invokes joints controlling larger arm segments, might be a contributing factor for the greater variability in results shown by S3’s inter-average DOS data and in the lower quality assessments by the adjudicators.

Regarding left-hand grouping, five of the six subjects employed a methodical strategy organized through the use of a guide finger. This is an entrained strategy that has been advocated in treatises for more than two hundred years. Given the findings of the current study—that DOS tends to be discrete for each performer—grouping becomes an indispensable strategy at faster tempi. For example, given that S6’s DOS is about 450 ms, and the fastest tempo required that she played 5 notes per second, the initiation of her shift had to occur three notes earlier than onsets of the arrival note of the shift. This cannot be accomplished using a note-to-note playing process. In action-by-action playing, the somatosensory role of the guide finger during shifting is greatly reduced as the finger ending the shift is typically placed onto the string prior to achieving an optimal hand and arm orientation for the new position. Only one subject (S3) used action-by-action finger control. EST variability in his performance and his overall lower adjudication scores suggest this strategy to be less effective than grouping.

During training, violinists spend countless hours exploring alternative motor control sequences in order to synchronize elements of performance. Since shifting involves asymmetrical coordination of multiple joints in the kinematic chains leading to the fingers, stabilization of one or more control elements should theoretically simplify skill complexity and contributing to increased accuracy (Magill, 2001). Our data suggests that optimization of shifting involves minimizing tempo-dependent effects on the execution of the skill. With speed relegated to a quasi-constant in the shifting “equation,” general finger control patterning in the left hand and choice of shifting initiator dominate motor strategy selection; these become anthropometrically-influenced variables that may influence musical effects and artistic preferences in performance. By understanding the interaction of these variables, violinists should be able to accelerate skill acquisition, increase accuracy, and achieve a level of automation, leaving them more at liberty to focus on personalized interpretive outcomes.

The current study has several limitations. First, it is a case series of six highly trained violinists. As such, results may point to the connections discussed but cannot be said to conclusively determine them. Second, it is only circumstantial that the subject population had three males and three females. For the study to speculate on gender-related results, confounding variables (e.g., body height) would have needed to be controlled. Finally, the shifts observed in the current research were limited by the choice of music. The music only used shifts traversing an interval of a third—the most common shift distance used in violin playing. It may be that shifts of greater distances reveal additional grouping strategies. The current study sets a baseline for future work investigating shifting, a skill vital in violin performance. To fully unravel the mysteries of personal performance style, additional studies of larger subject populations will be needed.

## Conclusion

Motor control during instrumental music performance is extremely complex and evaluating its success in musical context necessitates consideration of the aural result. For the violinist, motor behavior involves entrainment of strategies to coordinate both gross and fine motor control, nuanced audial and neurosensory discrimination skills, and adaptation in a temporal endeavor where success or failure may be measurable in milliseconds while the conceptual integrity of a performance may unfold over hours. Shifting ranks among the most important of violinistic skills and, as such, provides an ideal task in which to begin to discuss how these concepts intersect.

During shifting, preorganization of motor behavior is requisite but adaptation to events of the moment is expected. For an expert performer, the richness of his/her timbral vocabulary is in great part dependent on interpretive choices associated with where, when, and how to employ shifting (Baillot, 1831 (transl. 1991 L Goldberg)). Like enunciation for the singing voice, the act of shifting results in subtle nuances of sound in between the pitches notated by the composer. Hence, in actual performing circumstances, the sounds of shifting are deliberately manipulated and must be understood as elements of artistry, not of execution. The use of a Kreutzer Etude in the current study moves one step closer to shifting skill evaluation in the context of actual performance. The Etude is conceived in a way that leaves little room for error in skill execution, making it useful in identifying underlying signature elements of motor control. Understanding shifting execution in this utilitarian setting in turn provides insight for future work that might examine how performers manipulate motor skills as part of an artistic process.

The aims of the current study were: (1) to better understand the skill of left-hand position changing (shifting) in terms that might make it easier to acquire and automate in the context of learning and performance, and (2) to initiate discussion on relationships between motor control and individuality of performance execution. Among our subjects, some elements of the skill were individualized in surprising ways while others were explainable by anthropometry, ergonomics and entrainment. Remarkably, with the exception of one subject results DOS to be independent of tempo for the speeds tested. Each of the violinists in the study appears to have developed a personalized pacing for shifts. With regard to control sequencing and motor organization, subjects with the most successful aural results all made methodical use of a guide finger during left hand motor control, a strategy instilled through long entrainment. Use of guide fingers facilitated grouping strategies whereby hand posture could be organized during shifting, aiding in the stabilization of overall timing effects. Individualization of motor organization was found by examining which part of the arm/hand initiated shifting. This parameter appears to be strongly related to anthropometry.

Scientific studies can play an important role in challenging the inertia of tradition that exists in the performing arts. Most importantly, they provide objective ground upon which to discuss elements of a performer’s “style” that might otherwise simply be identified as natural ability or “talent.” Individualization that maintains reference to existing musico-cultural traditions is widely recognizable as a hallmark of artistry. In instrumental music performance, an understanding of biomechanics, ergonomics, and the strategic use of motor behaviors can help explain the interaction of the artist with the tools of performance in the context of the desired musical outcome. Ultimately, these elements become manifest in subtleties of tone, timing and expression during performance which listeners experience as signature characteristics of the performer.

The study used methods from movement science to examine timing elements and motor control strategies during shifting, a skill vital in violin performance. It contributes to fundamental understanding of the skill and discusses elements of individualization among subjects in terms of anthropometry and the strategic use of motor behaviors developed through lengthy practice. Finally, it considers the implications of these in terms of the aural result. In doing so, the current study points in the direction of a research inquiry model that might meaningfully influence music pedagogy and provides a basis for future studies that examine the manipulation of motor behaviors as a foundational element of artistry in music performance.

## Supplemental Information

10.7717/peerj.1299/supp-1 Data S1 Supplemental files—raw data

10.7717/peerj.1299/supp-2 Supplemental Information 1 Supplemental files—statistical comparisons

10.7717/peerj.1299/supp-3 Supplemental Information 2 Supplemental files—assements of expert adjudicators

## Appendix A. Tuning Discrepancy in the Music Scale

Western music uses a scale in which octaves are divided into 12 parts. Octaves are defined as pitches where vibrational frequencies may be described as follows: NoteName =*f* × 2^*n*^ (where *f* is the starting fundamental frequency in Hz and *n* is an integer). Thus if the note “A” is defined as a pitch vibrating at 220 Hz, then pitches at vibrational frequencies of 55, 110, 440, 880, etc., are all identified as the note “A,” although they are said to occur in different octave registers. Notes named in this manner are identified as belonging to the same “pitch class set” (notes using the same name). Once an initial pitch has been defined, the remaining notes of the western chromatic scale may be determined in a process (documented by Pythagoras) whereby the frequency of vibration of a string (or the column of air in the case of a wind instrument) is altered by stopping the string (lifting fingers off the holes) at various fractional distances along its length. The interval of a perfect fifth is defined as the note that sounds when a string is stopped at a fractional ratio of 3:2 along its length. In theory, starting from a given fundamental note and repeating this process 11 times should give rise to all 12 notes of the western chromatic scale. The 12th repetition of the process should arrive back at the same pitch class set as the starting note (but seven octave registers higher). For example, taking *f* = 50 Hz as starting pitch, the process of moving seven octaves higher (50 Hz × 2^7^ = 6,400 Hz) should equal the process of moving twelve perfect fifths higher (50 Hz × (3/2)^12^ = 6,487.3 Hz). The difference (87.3 Hz, in this case) is an artifact of the western system; theory does not correspond to reality. This artifact requires that pitches be slightly modified to accommodate the error. For fixed-pitch instruments such as the piano, accommodation is typically achieved by dividing the difference relatively equally among all the different pitches. For instruments like the violin, varying accommodations are used depending on underlying harmonic structures.
