# Supplementary material for: Unraveling mysteries of personal performance style; biomechanics of left-hand position changes (shifting) in violin performance
Source: PeerJ. 2015 Oct 1;3:e1299. doi: 10.7717/peerj.1299 (PMC4636401; doi:10.7717/peerj.1299)
Supplement: Data S1 [file peerj-03-1299-s001.pdf]

Shifting patterns identified

Shifting Patterns	shift numer	Start Finger	End finger	Start pos	End pos	String Crossing (Y/n)	Bow Direction Change (Y/N)	String used
I	1,7,13	2	4	1	3	y	n	e,a
II	2,8	2	4	3	5	n	n	a
III	3,9	2	4	5	3	n	n	a
IV	4,6,10,12,18	1	3	3	1	n	n	a
V	5,11	1	3	1	3	n	n	a
VI	14	2	4	3	1	n	n	a
VII	15	2	4	1	3	y	n	a,d
VIII	16	1	3	3	1	n	n	e
IX	17	1	3	1	3	y	n	e,a

Female Subjects:

S4

Tempo 60 a

Shifting Patterns	shift #	Begin	tme interval between shift beginnings (s)	error in shift beginnig accuracy (s)		End	time interval between shift endings	error in shift ending accuracy (s)	initiator	Number of frames at 200 f/s	
A	1	468	1.02	0.01		541	1.03	0.02	W	73	
B	2	671	0.98	0.03		746	0.95	0.05	W	75	
C	3	866	2.11	0.05		936	2.13	0.06	T	70	
D	4	1288	0.99	0.02		1361	0.98	0.02	T	73	
E	5	1485	1.01	0.00		1557	1.02	0.01	W	72	
D	6	1686	1.94	0.03		1760	1.94	0.06	W	74	
A	7	2074	1.04	0.04		2147	1.10	0.10	W	73	
B	8	2282	1.01	0.01		2366	0.97	0.03	W	84	
C	9	2484	1.94	0.03		2560	1.92	0.09	T	76	
D	10	2872	1.05	0.05		2943	1.04	0.04	T	71	
E	11	3082	0.99	0.02		3151	0.98	0.02	W	69	
D	12	3279	2.04	0.02		3347	2.01	0.00	W	68	
A	13	3686	0.96	0.05		3748	1.01	0.01	W	62	
F	14	3877	0.99	0.01		3950	0.99	0.01	T	73	
G	15	4075	1.99	0.01		4148	1.99	0.01	W	73	
H	16	4472	1.02	0.02		4545	1.03	0.02	T	73	
I	17	4676	0.96	0.04		4750	0.95	0.06	W	74	
D	18	4868				4939			T	71	
				0.03	s			0.04	s	72.4	f
				0.02	s			0.03	s	4.3	f

Tempo 60 b

Begin	tme interval between shift beginnings (s)	error in shift beginnig accuracy (s)	End	time interval between shift endings	error in shift ending accuracy (s)	initiator	Number of frames at 200 f/s
570	0.98	0.02	641	0.99	0.01	W	71
766	0.99	0.02	839	0.99	0.02	W	73
963	1.97	0.02	1036	1.97	0.02	T	73
1357	1.02	0.02	1429	0.99	0.02	T	72
1561	0.97	0.04	1626	1.00	0.01	W	65
1754	1.99	0.01	1825	2.04	0.04	T	71
2152	1.07	0.07	2233	1.04	0.04	W	81
2366	0.98	0.03	2441	0.98	0.03	W	75
2561	2.01	0.00	2636	2.00	0.00	T	75

2962	0.99	0.01		3036	0.99	0.02	T		74	
3160	1.00	0.01		3233	0.99	0.01	W		73	
3359	2.00	0.00		3431	2.00	0.00	T		72	
3758	0.98	0.02		3831	0.99	0.01	W		73	
3954	1.06	0.06		4029	1.05	0.05	T		75	
4166	1.98	0.01		4239	1.99	0.01	W		73	
4561	1.01	0.00		4636	0.99	0.01	T		75	
4762	0.97	0.04		4834	0.98	0.02	W		72	
4955				5030			T		75	
		0.02	s			0.02	s		73.2	f
		0.02	s			0.01	s		3.1	f

End accur.	27	ms	Duration	364	ms
StDev	21	ms	StDev	18	ms

Note: the Duration is converted from f (frames) to ms by timing 5 (1s/200Hz=5ms).

Tempo 72 a

Begin	tme interval between shift beginnings (s)	error in shift beginnig accuracy (s)		End	time interval between shift endings	error in shift ending accuracy (s)	initiator	Number of frames at 200 f/s	
778	1.01	0.01		841	1.08	0.08	W	63	
947	1.03	0.03		1021	1.05	0.05	W	74	
1118	2.05	0.02		1196	2.11	0.06	T	78	
1459	1.01	0.01		1548	0.92	0.08	T	89	
1627	0.94	0.06		1702	0.93	0.07	W	75	
1783	1.93	0.04		1857	1.94	0.06	T	74	
2104	1.08	0.08		2181	1.04	0.04	W	77	
2284	0.95	0.05		2354	0.96	0.04	W	70	
2443	1.97	0.02		2514	1.98	0.02	T	71	
2771	0.99	0.01		2844	1.02	0.02	T	73	
2936	0.98	0.02		3014	0.94	0.06	W	78	
3099	2.03	0.01		3171	2.04	0.04	T	72	
3437	0.99	0.01		3511	1.04	0.04	W	74	
3602	1.01	0.01		3684	0.98	0.02	T	82	
3771	1.97	0.02		3848	1.95	0.05	W	77	
4099	1.03	0.03		4173	1.04	0.04	T	74	
4271	1.06	0.06		4346	1.06	0.06	W	75	
4448				4523			T	75	
		0.03	s			0.05	s	75.1	f
		0.02	s			0.02	s	5.3	f

Tempo 72 b

Begin	tme interval between shift beginnings (s)	error in shift beginnig accuracy (s)	End	time interval between shift endings	error in shift ending accuracy (s)	initiator	Number of frames at 200 f/s
502	1.07	0.07	576	1.07	0.07	W	74
681	0.92	0.08	755	0.94	0.06	W	74
834	1.97	0.01	911	1.96	0.02	T	77
1163	1.03	0.03	1238	1.04	0.04	T	75
1334	0.97	0.03	1411	0.95	0.05	W	77
1496	2.08	0.04	1570	2.05	0.05	T	74
1843	0.97	0.03	1912	1.01	0.01	W	69
2005	1.01	0.01	2080	0.95	0.05	W	75
2173	1.97	0.01	2239	2.02	0.02	T	66
2502	0.99	0.01	2575	1.00	0.00	T	73
2667	0.94	0.06	2742	1.01	0.01	W	75
2823	2.02	0.01	2911	1.97	0.03	T	88
3159	1.07	0.07	3240	1.04	0.04	W	81

3338	0.99	0.01		3413	0.98	0.02	T		75	
3503	1.96	0.02		3577	1.96	0.04	W		74	
3829	1.04	0.04		3904	1.04	0.04	T		75	
4002	1.01	0.01		4077	1.03	0.03	W		75	
4171				4248			T		77	
		0.03	s			0.03	s		75.2	f
		0.03	s			0.02	s		4.5	f

End accur.	40	ms	Duration	376	ms
StDev	19	ms	StDev	24	ms

Tempo 100

Begin	tme interval between shift beginnings (s)	error in shift beginnig accuracy (s)		End	time interval between shift endings	error in shift ending accuracy (s)	initiator	Number of frames at 200 f/s	
621	1.08	0.08		716	0.93	0.07	W	95	
750	0.97	0.03		828	0.90	0.10	W	78	
866	1.90	0.05		936	1.91	0.05	T	70	
1094	1.02	0.02		1165	1.03	0.02	T	71	
1216	1.07	0.07		1288	0.90	0.10	W	72	
1344	1.93	0.07		1396	2.05	0.05	T	52	
1576	1.08	0.08		1642	1.12	0.12	W	66	
1705	0.94	0.06		1776	0.94	0.06	W	71	
1818	2.04	0.04		1889	2.03	0.03	T	71	
2063	0.99	0.01		2133	0.99	0.01	T	70	
2182	1.03	0.02		2252	1.04	0.04	W	70	
2305	2.01	0.01		2377	1.99	0.01	T	72	
2546	1.13	0.13		2616	1.03	0.02	W	70	
2681	0.93	0.07		2739	0.99	0.01	T	58	
2793	2.00	0.00		2858	2.04	0.04	W	65	
3033	0.97	0.03		3103	0.98	0.03	T	70	
3149	1.03	0.03		3220	1.03	0.02	W	71	
3273				3343			T	70	
		0.05	s			0.05	s	70.1	f
		0.03	s			0.03	s	8.4	f

End Accur.	46	Duration	351	ms
StDev	33	StDev	42	ms

S5

Tempo 60 a

Begin	tme interval between shift beginnings (s)	error in shift beginnig accuracy (s)		End	time interval between shift endings	error in shift ending accuracy (s)	initiator	Number of frames at 200 f/s	
650	1.01	0.01		706	0.95	0.05	T	56	
852	0.89	0.11		896	0.98	0.03	T	44	
1030	1.96	0.02		1091	2.05	0.02	T	61	
1422	1.07	0.07		1501	0.99	0.02	T	79	
1636	1.10	0.10		1698	0.99	0.01	W	62	
1856	2.03	0.01		1896	2.09	0.09	T	40	
2262	0.98	0.02		2313	0.93	0.07	T	51	
2458	0.98	0.03		2499	1.03	0.02	T	41	
2653	1.97	0.02		2704	1.99	0.01	T	51	
3046	0.96	0.04		3102	0.98	0.03	T	56	
3238	0.96	0.04		3297	0.99	0.02	W	59	
3430	1.99	0.01		3494	2.07	0.06	T	64	
3828	0.98	0.03		3907	0.95	0.06	T	79	
4023	1.03	0.03		4096	1.01	0.01	T	73	
4229	2.02	0.01		4298	2.01	0.00	W	69	
4632	1.03	0.03		4699	0.99	0.02	T	67	
4838	1.00	0.00		4896	1.03	0.02	W	58	
5038				5101			T	63	
		0.03	s			0.03	s	59.6	f
		0.03	s			0.02	s	11.5	f

Tempo 60 b

Begin	tme interval between shift beginnings (s)	error in shift beginnig accuracy (s)	End	time interval between shift endings	error in shift ending accuracy (s)	initiator	Number of frames at 200 f/s
324	0.98	0.03	380	0.95	0.06	T	56
519	0.96	0.04	569	1.05	0.05	T	50
711	2.04	0.02	779	2.09	0.04	T	68
1118	1.07	0.06	1196	0.99	0.01	T	78
1331	1.05	0.05	1394	1.02	0.02	T	63
1541	2.04	0.02	1598	1.98	0.02	T	57
1949	0.99	0.01	1994	0.97	0.03	T	45
2147	0.97	0.03	2188	1.01	0.00	T	41
2341	1.96	0.02	2389	2.03	0.03	T	48

2733	1.03	0.02		2795	0.99	0.01	T	62	
2938	0.98	0.02		2993	0.99	0.01	T	55	
3134	1.96	0.02		3191	1.99	0.01	T	57	
3525	0.97	0.03		3589	1.00	0.01	T	64	
3719	0.95	0.06		3788	0.99	0.01	T	69	
3908	2.01	0.00		3986	1.96	0.04	W	78	
4309	1.04	0.03		4378	0.99	0.01	T	69	
4516	0.96	0.05		4576	1.05	0.05	W	60	
4707				4786			T	79	
		0.03	s			0.02	s	61.1	f
		0.02	s			0.02	s	11.2	f

End accur.	28	ms	Duration	302	ms
StDev	21	ms	StDev	57	ms

Tempo 72 a

Begin	tme interval between shift beginnings (s)	error in shift beginnig accuracy (s)		End	time interval between shift endings	error in shift ending accuracy (s)	initiator	Number of frames at 200 f/s	
729	0.97	0.03		797	0.89	0.11	T	68	
891	1.03	0.03		946	1.00	0.00	T	55	
1063	2.03	0.01		1113	2.15	0.07	T	50	
1401	1.01	0.01		1471	0.97	0.03	T	70	
1569	1.03	0.03		1633	1.10	0.10	T	64	
1740	2.05	0.02		1816	1.94	0.06	T	76	
2081	1.01	0.01		2140	1.00	0.00	T	59	
2250	1.00	0.00		2306	1.04	0.04	T	56	
2417	2.02	0.01		2479	1.99	0.01	T	62	
2753	0.98	0.02		2810	1.00	0.00	T	57	
2917	1.01	0.01		2976	1.03	0.03	T	59	
3085	1.93	0.04		3147	1.97	0.03	T	62	
3406	0.97	0.03		3476	0.89	0.11	T	70	
3568	1.01	0.01		3624	1.06	0.06	T	56	
3736	1.99	0.01		3800	1.93	0.07	T	64	
4067	0.99	0.01		4121	1.02	0.02	T	54	
4232	0.98	0.02		4291	1.00	0.00	T	59	
4396				4458			T	62	
		0.02	s			0.04	s	61.3	f
		0.01	s			0.04	s	6.6	f

72b

Begin	tme interval between shift beginnings (s)	error in shift beginnig accuracy (s)	End	time interval between shift endings	error in shift ending accuracy (s)	initiator	Number of frames at 200 f/s
521	0.95	0.05	582	0.91	0.09	T	61
679	0.98	0.02	734	1.00	0.00	T	55
843	2.08	0.04	900	2.12	0.06	T	57
1189	0.97	0.03	1253	0.99	0.01	T	64
1351	1.00	0.00	1418	1.04	0.04	T	67
1517	2.05	0.03	1592	2.00	0.00	T	75
1859	0.98	0.02	1926	0.92	0.08	T	67
2023	1.01	0.01	2080	1.03	0.03	T	57
2192	2.00	0.00	2252	2.01	0.01	T	60
2526	0.99	0.01	2587	0.91	0.09	T	61
2691	0.95	0.05	2738	1.01	0.01	W	47

2850	1.94	0.03		2906	1.95	0.05	T		56	
3173	0.97	0.03		3231	1.00	0.00	T		58	
3335	0.96	0.04		3398	0.97	0.03	T		63	
3495	2.01	0.00		3560	2.03	0.03	T		65	
3830	1.03	0.03		3899	1.06	0.06	T		69	
4001	0.95	0.05		4076	0.88	0.12	W		75	
4160				4222			T		62	
		0.03	s			0.04	s		62.2	f
		0.02	s			0.04	s		7.0	f

End accur.	43	ms	Duration	309	ms
StDev	37	ms	StDev	34	ms

100

Begin	tme interval between shift beginnings (s)	error in shift beginnig accuracy (s)		End	time interval between shift endings	error in shift ending accuracy (s)	initiator	Number of frames at 200 f/s	
576	1.03	0.02		635	0.98	0.03	T	59	
699	1.02	0.02		752	1.03	0.03	T	53	
821	2.08	0.04		876	2.03	0.01	T	55	
1070	1.08	0.08		1119	1.07	0.07	T	49	
1199	0.96	0.04		1247	1.03	0.02	T	48	
1314	2.05	0.05		1370	2.09	0.09	T	56	
1560	0.98	0.03		1621	0.98	0.03	T	61	
1677	0.96	0.04		1738	0.98	0.02	T	61	
1792	1.99	0.01		1856	1.98	0.02	T	64	
2031	0.97	0.03		2094	0.99	0.01	T	63	
2147	0.99	0.01		2213	0.97	0.03	T	66	
2266	2.01	0.01		2329	2.09	0.09	T	63	
2507	1.03	0.03		2580	0.98	0.02	T	73	
2631	1.04	0.04		2698	1.07	0.07	T	67	
2756	2.08	0.08		2826	2.04	0.04	T	70	
3005	1.01	0.01		3071	0.98	0.02	T	66	
3126	1.00	0.00		3189	0.98	0.03	W	63	
3246				3306			T	60	
		0.03	s			0.04	s	60.9	f
		0.02	s			0.03	s	6.8	f

End Accur.	36	ms	Duration	305	ms
StDev	27	ms	StDev	34	ms

S6

60a

Begin	tme interval between shift beginnings (s)	error in shift beginnig accuracy (s)		End	time interval between shift endings	error in shift ending accuracy (s)	initiator	Number of frames at 200 f/s	
824	1.09	0.09		924	0.95	0.06	T	100	
1041	0.90	0.10		1113	1.01	0.01	E	72	
1221	1.94	0.03		1315	1.93	0.04	T	94	
1609	1.13	0.13		1701	1.13	0.13	T	92	
1834	1.01	0.00		1927	0.99	0.01	T	93	
2035	2.04	0.02		2125	2.08	0.04	T	90	
2443	1.08	0.08		2540	1.00	0.01	T	97	
2658	1.03	0.02		2739	1.05	0.04	E	81	
2863	1.75	0.13		2948	1.85	0.08	T	85	
3213	1.08	0.08		3317	1.01	0.00	T	104	
3428	0.97	0.04		3518	1.03	0.03	T	90	
3621	2.04	0.02		3724	2.04	0.02	T	103	
4029	0.96	0.04		4132	0.93	0.08	T	103	
4221	1.04	0.03		4317	1.06	0.05	T	96	
4428	2.02	0.01		4528	1.94	0.03	T	100	
4831	1.05	0.04		4916	1.01	0.01	T	85	
5040	0.86	0.15		5118	0.95	0.05	T	78	
5211				5308			T	97	
		0.06	s			0.04	s	92.2	f
		0.04	s			0.03	s	9.1	f

60b

Begin	tme interval between shift beginnings (s)	error in shift beginnig accuracy (s)	End	time interval between shift endings	error in shift ending accuracy (s)	initiator	Number of frames at 200 f/s
443	1.09	0.09	539	1.01	0.00	T	96
661	1.00	0.00	740	1.06	0.05	E	79
861	2.05	0.02	951	2.04	0.02	T	90
1271	1.05	0.04	1359	1.04	0.04	T	88
1480	0.93	0.07	1567	0.96	0.04	T	87
1666	1.98	0.01	1759	2.02	0.01	T	93
2061	1.10	0.10	2162	1.01	0.00	T	101
2281	0.96	0.05	2363	0.98	0.02	E	82
2472	1.85	0.08	2559	1.91	0.05	T	87

2841	1.05	0.05		2940	1.04	0.04	T	99	
3051	0.93	0.08		3148	0.94	0.06	T	97	
3236	2.00	0.00		3336	2.03	0.01	T	100	
3636	1.08	0.08		3741	0.99	0.02	T	105	
3851	0.96	0.05		3938	0.99	0.02	T	87	
4042	1.99	0.01		4135	2.03	0.01	T	93	
4439	1.12	0.12		4541	1.02	0.01	T	102	
4662	0.97	0.03		4744	1.01	0.00	T	82	
4856				4945			T	89	
		0.05	s			0.02	s	92.1	f
		0.04	s			0.02	s	7.6	f

End accur.	32	ms	Duration	461	ms
StDev	26	ms	StDev	42	ms

72a

Begin	tme interval between shift beginnings (s)	error in shift beginnig accuracy (s)		End	time interval between shift endings	error in shift ending accuracy (s)	initiator	Number of frames at 200 f/s	
414	1.12	0.12		508	1.02	0.02	T	94	
601	0.95	0.05		678	1.03	0.03	E	77	
759	1.82	0.09		849	1.90	0.05	T	90	
1063	1.12	0.12		1165	1.04	0.04	T	102	
1250	0.93	0.07		1339	0.94	0.06	T	89	
1405	1.99	0.00		1496	1.99	0.00	T	91	
1737	1.04	0.04		1828	0.97	0.03	T	91	
1910	1.03	0.03		1990	1.07	0.07	E	80	
2081	1.83	0.09		2169	1.88	0.06	T	88	
2386	1.06	0.06		2483	1.05	0.05	T	97	
2562	0.97	0.03		2658	0.97	0.03	T	96	
2723	2.03	0.01		2819	2.03	0.02	T	96	
3061	1.03	0.03		3158	0.97	0.03	T	97	
3233	1.07	0.07		3320	1.07	0.07	T	87	
3411	1.96	0.02		3498	1.99	0.01	T	87	
3737	1.10	0.10		3829	1.01	0.01	T	92	
3921	0.82	0.18		3997	0.97	0.03	T	76	
4058				4158			T	100	
		0.07	s			0.04	s	90.6	f
		0.05	s			0.02	s	7.4	f

72b

Begin	tme interval between shift beginnings (s)	error in shift beginnig accuracy (s)	End	time interval between shift endings	error in shift ending accuracy (s)	initiator	Number of frames at 200 f/s
453	1.19	0.19	548	1.03	0.03	T	95
651	0.83	0.17	719	0.96	0.04	E	68
790	1.87	0.06	879	1.95	0.03	T	89
1102	1.19	0.19	1204	1.05	0.05	T	102
1300	0.86	0.14	1379	0.95	0.05	T	79
1443	2.00	0.00	1538	1.95	0.03	T	95
1777	0.98	0.02	1863	1.03	0.03	T	86
1941	1.15	0.15	2034	1.05	0.05	E	93
2132	1.85	0.08	2209	1.91	0.04	T	77
2440	1.03	0.03	2528	1.03	0.03	T	88
2611	0.83	0.17	2699	0.91	0.09	T	88

2749	2.07	0.03		2851	2.06	0.03	T	102	
3094	0.98	0.02		3195	1.02	0.02	T	101	
3257	1.09	0.09		3365	0.98	0.02	T	108	
3439	1.98	0.01		3528	2.00	0.00	T	89	
3769	1.07	0.07		3862	1.02	0.02	T	93	
3948	0.89	0.11		4032	0.97	0.03	T	84	
4097				4193			T	96	
		0.09	s			0.03	s	90.7	f
		0.07	s			0.02	s	9.9	f

End accur.	35	ms	Duration	453	ms
StDev	20	ms	StDev	43	ms

100

Begin	tme interval between shift beginnings (s)	error in shift beginnig accuracy (s)		End	time interval between shift endings	error in shift ending accuracy (s)	initiator	Number of frames at 200 f/s	
334	1.02	0.02		429	0.90	0.10	W	95	
456	0.99	0.01		537	1.09	0.09	E	81	
575	1.98	0.01		668	2.00	0.00	T	93	
813	1.19	0.19		908	1.08	0.08	T	95	
956	0.96	0.04		1037	1.01	0.01	W	81	
1071	1.84	0.08		1158	1.96	0.02	T	87	
1292	1.15	0.15		1393	0.93	0.07	W	101	
1430	0.90	0.10		1505	1.03	0.03	E	75	
1538	2.03	0.01		1629	2.00	0.00	W	91	
1781	0.98	0.02		1869	1.06	0.06	T	88	
1899	1.03	0.03		1996	0.93	0.07	W	97	
2023	1.82	0.09		2108	1.99	0.00	T	85	
2241	1.28	0.28		2347	1.09	0.09	W	106	
2395	0.78	0.22		2478	0.92	0.08	T	83	
2489	2.11	0.05		2588	2.08	0.04	T	99	
2742	0.85	0.15		2838	0.90	0.10	W	96	
2844	1.10	0.10		2946	0.95	0.05	W	102	
2976				3060			T	84	
		0.09	s			0.05	s	91.1	f
		0.08	s			0.04	s	8.5	f

End Accur.	52	ms	Duration	455	ms
StDev	36	ms	StDev	43	ms

Male Subjects:

S1

60a

Begin	tme interval between shift beginnings (s)	error in shift beginnig accuracy (s)		End	time interval between shift endings	error in shift ending accuracy (s)	initiator	Number of frames at 200 f/s	
159	0.94	0.06		216	0.98	0.03	W	57	
347	0.96	0.05		411	0.97	0.04	W	64	
538	1.93	0.04		604	1.98	0.01	W	66	
923	1.06	0.05		999	1.05	0.05	T	76	
1134	1.02	0.02		1209	1.00	0.01	W	75	
1338	1.94	0.03		1408	1.97	0.02	T	70	
1726	1.10	0.10		1801	1.03	0.03	W	75	
1946	1.08	0.08		2007	1.04	0.04	W	61	
2162	1.80	0.10		2215	1.92	0.04	W	53	
2522	1.02	0.02		2599	1.04	0.04	T	77	
2726	0.99	0.01		2807	0.93	0.07	W	81	
2924	2.03	0.01		2993	2.01	0.00	T	69	
3330	1.00	0.01		3395	0.97	0.03	W	65	
3529	0.94	0.06		3589	1.00	0.01	T	60	
3717	2.04	0.02		3788	1.99	0.01	W	71	
4125	1.01	0.00		4186	1.06	0.05	T	61	
4326	1.01	0.00		4397	0.97	0.04	W	71	
4527				4590			T	63	
		0.04	s			0.03	s	67.5	f
		0.03	s			0.02	s	7.6	f

60b

Begin	tme interval between shift beginnings (s)	error in shift beginnig accuracy (s)	End	time interval between shift endings	error in shift ending accuracy (s)	initiator	Number of frames at 200 f/s
446	0.95	0.05	512	0.91	0.10	W	66
636	1.03	0.02	693	0.96	0.05	W	57
841	1.98	0.01	884	2.06	0.03	W	43
1237	1.02	0.02	1296	1.05	0.04	T	59
1441	0.99	0.02	1505	0.94	0.06	W	64
1638	1.99	0.01	1692	2.03	0.01	T	54
2035	1.10	0.10	2097	1.02	0.01	W	62
2255	0.96	0.05	2300	0.99	0.02	W	45

2446	1.95	0.03		2497	1.96	0.02	W	51	
2836	0.98	0.02		2888	1.05	0.05	T	52	
3032	1.00	0.00		3098	0.95	0.05	W	66	
3232	1.99	0.01		3288	2.07	0.03	T	56	
3630	1.04	0.03		3702	1.02	0.02	W	72	
3837	0.94	0.06		3906	0.93	0.07	T	69	
4025	2.02	0.01		4092	1.95	0.03	W	67	
4429	1.02	0.01		4482	1.10	0.10	T	53	
4632	1.00	0.01		4701	0.98	0.03	W	69	
4831				4896			T	65	
		0.03	s			0.04	s	59.4	f
		0.03	s			0.03	s	8.5	f

End accur.	36	ms	Duration	317	ms
StDev	23	ms	StDev	40	ms

72a

Begin	tme interval between shift beginnings (s)	error in shift beginnig accuracy (s)		End	time interval between shift endings	error in shift ending accuracy (s)	initiator	Number of frames at 200 f/s	
342	1.07	0.07		410	1.01	0.01	W	68	
520	1.09	0.09		578	1.03	0.03	W	58	
701	1.69	0.15		749	1.80	0.10	W	48	
983	1.11	0.11		1049	1.03	0.03	T	66	
1168	0.89	0.11		1221	1.00	0.00	W	53	
1317	1.99	0.00		1387	2.00	0.00	T	70	
1649	1.02	0.02		1721	0.99	0.01	W	72	
1819	1.19	0.19		1886	1.09	0.09	W	67	
2017	1.65	0.18		2067	1.78	0.11	W	50	
2292	1.07	0.07		2363	1.06	0.06	T	71	
2471	1.00	0.00		2540	0.97	0.03	W	69	
2637	2.02	0.01		2701	2.11	0.06	T	64	
2974	1.10	0.10		3053	1.00	0.00	W	79	
3158	0.95	0.05		3219	1.00	0.00	T	61	
3316	2.04	0.02		3386	2.01	0.00	W	70	
3656	1.01	0.01		3721	1.01	0.01	T	65	
3825	1.02	0.02		3889	1.00	0.00	W	64	
3995				4056			T	61	
		0.07	s			0.03	s	64.2	f
		0.06	s			0.04	s	8.0	f

72b

Begin	tme interval between shift beginnings (s)	error in shift beginnig accuracy (s)	End	time interval between shift endings	error in shift ending accuracy (s)	initiator	Number of frames at 200 f/s
308	1.10	0.10	373	1.10	0.10	W	65
491	1.12	0.12	556	1.03	0.03	W	65
678	1.84	0.08	728	1.89	0.06	W	50
985	1.03	0.03	1043	1.05	0.05	T	58
1156	0.97	0.03	1218	0.97	0.03	W	62
1317	2.04	0.02	1379	2.02	0.01	T	62
1657	1.04	0.04	1715	1.01	0.01	W	58
1830	1.10	0.10	1883	1.06	0.06	W	53
2013	1.76	0.12	2059	1.85	0.07	W	46
2307	0.98	0.02	2368	1.00	0.00	T	61
2471	0.89	0.11	2535	0.94	0.06	W	64

2619	2.14	0.07	2692	2.04	0.02	T	73	
2975	1.07	0.07	3032	1.06	0.06	W	57	
3153	0.92	0.08	3209	0.95	0.05	T	56	
3306	2.02	0.01	3368	1.96	0.02	W	62	
3642	0.99	0.01	3694	1.10	0.10	T	52	
3807	1.02	0.02	3877	0.89	0.11	W	70	
3977			4026			T	49	
		0.06 s			0.05	s	59.1	f
		0.04 s			0.03	s	7.3	f

End accur.	41	ms	Duration	308	ms
StDev	35	ms	StDev	38	ms

100

Begin	tme interval between shift beginnings (s)	error in shift beginnig accuracy (s)		End	time interval between shift endings	error in shift ending accuracy (s)		initiator	Number of frames at 200 f/s	
237	1.07	0.07		296	1.03	0.02		W	59	
365	0.99	0.01		419	0.99	0.01		W	54	
484	1.81	0.10		538	1.84	0.08		W	54	
701	0.99	0.01		759	1.08	0.08		T	58	
820	1.10	0.10		888	1.00	0.00		W	68	
952	1.99	0.00		1008	2.08	0.04		T	56	
1191	1.11	0.11		1257	1.08	0.08		W	66	
1324	0.98	0.02		1386	0.92	0.08		W	62	
1442	1.79	0.10		1496	1.93	0.03		W	54	
1657	1.04	0.04		1728	1.03	0.02		T	71	
1782	1.12	0.12		1851	1.01	0.01		W	69	
1916	2.04	0.02		1972	2.01	0.00		T	56	
2161	1.01	0.01		2213	1.03	0.02		W	52	
2282	0.83	0.18		2336	0.90	0.10		T	54	
2381	2.18	0.09		2444	2.12	0.06		W	63	
2643	0.98	0.02		2698	1.07	0.07		T	55	
2761	1.02	0.02		2826	1.01	0.01		W	65	
2883				2947				T	64	
		0.06	s			0.04	s		60.0	f
		0.05	s			0.03	s		6.1	f

End Accur.	42	ms	Duration	300	ms
StDev	33	ms	StDev	30	ms

S2

60a

Begin	tme interval between shift beginnings (s)	error in shift beginnig accuracy (s)		End	time interval between shift endings	error in shift ending accuracy (s)	initiator	Number of frames at 200 f/s	
447	1.10	0.10		531	1.04	0.04	T	84	
666	1.08	0.08		739	1.07	0.06	T	73	
882	1.90	0.05		952	1.98	0.01	T	70	
1262	1.08	0.08		1348	1.03	0.03	T	86	
1477	0.89	0.11		1554	0.99	0.01	W	77	
1655	2.10	0.05		1752	2.05	0.02	T	97	
2074	1.00	0.00		2162	0.94	0.06	W	88	
2274	0.89	0.12		2350	0.97	0.03	T	76	
2451	2.08	0.04		2544	2.04	0.02	T	93	
2866	0.92	0.08		2951	0.99	0.01	T	85	
3050	1.04	0.03		3149	1.03	0.03	W	99	
3257	2.11	0.05		3355	2.00	0.00	T	98	
3678	0.98	0.02		3754	0.96	0.05	W	76	
3874	1.10	0.10		3945	1.05	0.05	T	71	
4093	1.86	0.07		4155	1.94	0.03	T	62	
4465	1.08	0.08		4542	1.04	0.04	T	77	
4680	0.95	0.06		4750	1.03	0.03	W	70	
4869				4956			T	87	
		0.06	s			0.03	s	81.6	f
		0.03	s			0.02	s	10.8	f

60b

Begin	tme interval between shift beginnings (s)	error in shift beginnig accuracy (s)	End	time interval between shift endings	error in shift ending accuracy (s)	initiator	Number of frames at 200 f/s
471	1.02	0.01	553	1.00	0.01	T	82
674	1.07	0.07	752	0.99	0.01	T	78
888	1.81	0.10	950	1.95	0.03	T	62
1249	1.14	0.14	1339	1.03	0.02	T	90
1476	0.82	0.18	1544	1.00	0.01	W	68
1640	2.18	0.09	1743	2.09	0.04	T	103
2075	1.03	0.02	2160	0.95	0.06	W	85
2280	0.82	0.19	2349	0.95	0.06	T	69
2443	2.17	0.08	2538	2.13	0.06	T	95

2876	0.99	0.02		2963	1.01	0.00	W	87	
3073	0.93	0.07		3164	0.95	0.05	W	91	
3259	2.06	0.03		3354	1.94	0.03	T	95	
3670	0.98	0.03		3742	0.94	0.06	W	72	
3865	1.08	0.08		3930	1.11	0.11	T	65	
4081	1.94	0.03		4151	1.99	0.01	T	70	
4469	1.02	0.02		4549	0.97	0.03	T	80	
4673	0.96	0.04		4743	1.01	0.00	W	70	
4865				4944			T	79	
		0.07	s			0.03	s	80.1	f
		0.05	s			0.03	s	11.8	f

End accur.	33	ms	Duration	404	ms
StDev	23	ms	StDev	57	ms

72a

Begin	tme interval between shift beginnings (s)	error in shift beginnig accuracy (s)	End	time interval between shift endings	error in shift ending accuracy (s)	initiator	Number of frames at 200 f/s	
323	1.06	0.06	407	0.95	0.05	T	84	
500	0.97	0.03	565	1.00	0.00	T	65	
661	1.80	0.10	731	1.96	0.02	T	70	
961	1.13	0.13	1058	1.01	0.01	T	97	
1150	0.98	0.02	1227	1.09	0.09	W	77	
1313	2.17	0.09	1408	2.03	0.02	T	95	
1675	0.96	0.04	1747	0.92	0.08	W	72	
1835	0.77	0.23	1901	0.95	0.05	T	66	
1963	2.10	0.05	2060	2.05	0.02	T	97	
2313	0.91	0.09	2401	0.95	0.05	T	88	
2464	1.04	0.04	2559	1.01	0.01	W	95	
2638	2.14	0.07	2728	2.05	0.02	T	90	
2994	0.86	0.14	3069	0.93	0.07	W	75	
3138	1.09	0.09	3224	1.05	0.05	T	86	
3320	1.85	0.07	3399	1.95	0.03	T	79	
3629	1.24	0.24	3724	1.04	0.04	T	95	
3835	0.85	0.15	3898	0.98	0.02	W	63	
3977			4061			T	84	
		0.10	s		0.04	s	82.1	f
		0.06	s		0.02	s	11.7	f

72b

Begin	tme interval between shift beginnings (s)	error in shift beginnig accuracy (s)	End	time interval between shift endings	error in shift ending accuracy (s)	initiator	Number of frames at 200 f/s
277	1.06	0.06	358	0.98	0.02	T	81
453	0.86	0.14	521	1.00	0.00	T	68
596	1.97	0.01	688	1.97	0.02	T	92
925	1.03	0.03	1016	0.98	0.02	T	91
1097	1.01	0.01	1180	1.06	0.06	W	83
1265	2.15	0.07	1357	2.03	0.02	T	92
1623	1.00	0.00	1696	0.93	0.07	W	73
1789	0.82	0.18	1851	0.98	0.02	T	62
1926	2.01	0.00	2014	2.00	0.00	T	88
2261	0.96	0.04	2347	0.99	0.01	T	86
2421	1.02	0.02	2512	1.03	0.03	W	91

2591	2.11	0.05		2684	2.01	0.00	T	93	
2942	1.11	0.11		3019	0.98	0.02	W	77	
3127	0.98	0.02		3183	1.00	0.00	T	56	
3291	1.90	0.05		3350	1.99	0.01	T	59	
3608	1.01	0.01		3681	0.97	0.03	T	73	
3776	0.82	0.18		3842	1.01	0.01	W	66	
3912				4011			T	99	
		0.06	s			0.02	s	79.4	f
		0.06	s			0.02	s	13.2	f

End accur.	29	ms	Duration	404	ms
StDev	22	ms	StDev	62	ms

100

Begin	tme interval between shift beginnings (s)	error in shift beginnig accuracy (s)		End	time interval between shift endings	error in shift ending accuracy (s)	initiator	Number of frames at 200 f/s	
432	0.90	0.10		503	0.95	0.05	T	71	
540	1.07	0.07		617	0.96	0.04	T	77	
668	1.75	0.13		732	1.98	0.01	T	64	
878	1.10	0.10		969	0.95	0.05	T	91	
1010	0.88	0.13		1083	0.98	0.02	W	73	
1115	2.30	0.15		1201	2.22	0.11	T	86	
1391	0.96	0.04		1467	1.01	0.01	W	76	
1506	0.88	0.12		1588	0.88	0.12	T	82	
1612	2.13	0.06		1694	2.06	0.03	T	82	
1867	0.90	0.10		1941	0.94	0.06	T	74	
1975	0.88	0.13		2054	0.98	0.02	W	79	
2080	2.26	0.13		2172	2.11	0.05	T	92	
2351	0.93	0.07		2425	0.84	0.16	W	74	
2463	0.88	0.12		2526	1.01	0.01	T	63	
2569	2.03	0.01		2647	1.98	0.01	T	78	
2812	1.01	0.01		2885	0.99	0.01	T	73	
2933	0.83	0.18		3004	1.03	0.03	W	71	
3032				3128			T	96	
		0.10	s			0.05	s	77.9	f
		0.05	s			0.04	s	9.0	f

End Accur.	46	ms	Duration	389	ms
StDev	44	ms	StDev	45	ms

S6

60a

Begin	tme interval between shift beginnings (s)	error in shift beginnig accuracy (s)		End	time interval between shift endings	error in shift ending accuracy (s)	initiator	Number of frames at 200 f/s	
332	1.05	0.05		403	0.98	0.02	W	71	
542	0.99	0.01		599	1.01	0.01	E	57	
740	2.11	0.05		801	2.16	0.08	E	61	
1162	0.92	0.08		1233	0.99	0.02	T	71	
1346	0.93	0.07		1430	0.84	0.17	W	84	
1532	2.02	0.01		1597	2.03	0.03	T	65	
1935	1.13	0.13		2003	1.03	0.03	W	68	
2161	0.96	0.04		2209	1.02	0.02	E	48	
2353	1.96	0.02		2413	1.98	0.02	E	60	
2744	0.99	0.02		2809	0.99	0.01	T	65	
2941	1.02	0.02		3007	1.01	0.01	W	66	
3145	2.01	0.00		3209	2.03	0.03	T	64	
3546	0.96	0.04		3615	0.98	0.03	W	69	
3738	1.01	0.00		3810	0.96	0.04	T	72	
3939	2.17	0.09		4002	2.21	0.21	W	63	
4373	0.84	0.16		4443	0.82	0.19	T	70	
4541	1.02	0.02		4606	1.02	0.01	W	65	
4745				4809			T	64	
		0.05	s			0.05	s	65.7	f
		0.05	s			0.07	s	7.4	f

60b

Begin	tme interval between shift beginnings (s)	error in shift beginnig accuracy (s)	End	time interval between shift endings	error in shift ending accuracy (s)	initiator	Number of frames at 200 f/s
314	1.03	0.02	380	0.99	0.01	W	66
519	0.96	0.04	578	1.02	0.01	E	59
711	2.04	0.02	781	2.06	0.03	E	70
1118	1.12	0.12	1192	1.19	0.19	T	74
1341	0.90	0.10	1429	0.79	0.21	W	88
1521	2.14	0.07	1587	2.04	0.04	T	66
1949	0.89	0.11	1994	0.97	0.03	W	45
2127	0.97	0.03	2188	1.01	0.00	E	61
2321	2.01	0.00	2389	1.98	0.02	E	68

2723	0.85	0.15		2785	0.89	0.11	T	62	
2893	1.21	0.21		2963	1.18	0.18	W	70	
3134	1.96	0.02		3199	1.95	0.05	T	65	
3525	0.97	0.03		3589	1.00	0.01	W	64	
3719	0.95	0.06		3788	0.99	0.01	T	69	
3908	2.01	0.00		3986	1.96	0.04	W	78	
4309	1.00	0.00		4378	0.99	0.01	T	69	
4509	0.99	0.01		4576	1.03	0.02	W	67	
4707				4781			T	74	
		0.06	s			0.06	s	67.5	f
		0.06	s			0.07	s	8.8	f

End accur.	55	ms	Duration	333	ms
StDev	67	ms	StDev	40	ms

72a

Begin	tme interval between shift beginnings (s)	error in shift beginnig accuracy (s)		End	time interval between shift endings	error in shift ending accuracy (s)	initiator	Number of frames at 200 f/s	
566	0.99	0.01		629	0.97	0.03	W	63	
731	0.99	0.01		791	1.01	0.01	E	60	
896	2.09	0.04		959	2.09	0.05	E	63	
1244	1.01	0.01		1308	1.01	0.01	T	64	
1412	1.03	0.03		1476	1.21	0.21	W	64	
1583	2.05	0.02		1677	1.93	0.07	T	94	
1924	0.99	0.01		1998	0.94	0.06	W	74	
2089	1.03	0.03		2154	1.01	0.01	E	65	
2260	2.02	0.01		2322	1.99	0.01	E	62	
2596	0.98	0.02		2653	1.00	0.00	T	57	
2760	1.01	0.01		2819	1.01	0.01	W	59	
2928	1.93	0.04		2988	1.93	0.07	T	60	
3249	0.95	0.05		3309	0.95	0.05	W	60	
3407	0.99	0.01		3467	0.98	0.02	T	60	
3572	2.00	0.00		3630	2.03	0.03	W	58	
3906	1.01	0.01		3969	0.99	0.01	T	63	
4075	0.98	0.02		4134	1.00	0.00	W	59	
4239				4301			T	62	
		0.02	s			0.04	s	63.7	f
		0.01	s			0.05	s	8.4	f

72b

Begin	tme interval between shift beginnings (s)	error in shift beginnig accuracy (s)	End	time interval between shift endings	error in shift ending accuracy (s)	initiator	Number of frames at 200 f/s
464	0.97	0.03	524	0.95	0.05	W	60
625	0.96	0.04	683	0.98	0.02	E	58
785	2.11	0.05	847	2.12	0.06	E	62
1136	0.97	0.03	1200	1.01	0.01	T	64
1298	0.94	0.06	1369	1.02	0.02	W	71
1454	2.11	0.06	1539	1.99	0.01	T	85
1806	0.98	0.02	1871	0.96	0.04	W	65
1970	1.01	0.01	2031	1.01	0.01	E	61
2139	2.00	0.00	2199	2.04	0.04	E	60
2473	0.85	0.15	2539	0.81	0.19	T	66
2615	1.09	0.09	2674	1.03	0.03	W	59

2797	1.94	0.03		2846	1.99	0.01	T	49	
3120	0.97	0.03		3178	1.00	0.00	W	58	
3282	0.96	0.04		3345	0.97	0.03	T	63	
3442	2.01	0.00		3507	2.02	0.02	W	65	
3777	1.03	0.03		3843	1.02	0.02	T	66	
3948	0.96	0.04		4013	0.99	0.01	W	65	
4108				4178			T	70	
		0.04	s			0.03	s	63.7	f
		0.04	s			0.04	s	7.3	f

End accur.	36	ms	Duration	319	ms
StDev	46	ms	StDev	39	ms

100

Begin	tme interval between shift beginnings (s)	error in shift beginnig accuracy (s)		End	time interval between shift endings	error in shift ending accuracy (s)	initiator	Number of frames at 200 f/s	
297	1.03	0.02		356	0.98	0.03	W	59	
420	1.02	0.02		473	1.03	0.03	E	53	
542	2.11	0.05		597	2.03	0.01	E	55	
795	1.02	0.02		840	1.07	0.07	T	45	
917	0.98	0.02		968	1.03	0.02	W	51	
1035	2.05	0.05		1091	2.09	0.09	T	56	
1281	0.98	0.03		1342	0.92	0.08	W	61	
1398	1.00	0.00		1452	1.07	0.07	E	54	
1518	2.08	0.08		1580	2.13	0.13	E	62	
1767	0.89	0.11		1836	0.82	0.18	T	69	
1874	0.94	0.06		1934	0.97	0.03	W	60	
1987	2.08	0.08		2050	2.09	0.09	T	63	
2237	1.01	0.01		2301	0.99	0.01	W	64	
2358	0.99	0.01		2420	0.98	0.02	T	62	
2477	2.15	0.15		2538	2.12	0.12	W	61	
2735	0.93	0.07		2792	1.07	0.07	T	57	
2847	1.04	0.04		2920	0.89	0.11	W	73	
2972				3027			T	55	
		0.05	s			0.07	s	58.9	f
		0.04	s			0.05	s	6.6	f

End Accur.	68	ms	Duration	294	ms
StDev	49	ms	StDev	33	ms
