# Supplementary material for: Unraveling mysteries of personal performance style; biomechanics of left-hand position changes (shifting) in violin performance
Source: PeerJ. 2015 Oct 1;3:e1299. doi: 10.7717/peerj.1299 (PMC4636401; doi:10.7717/peerj.1299)
Supplement: Supplemental Information 1 [file peerj-03-1299-s002.pdf]

Multiple comparisons (SPSS v.16.0)

Accuracy

Female Subjects:

**ANOVA**

AccuracyS4

| | Sum of Squares | df | Mean Square | F | Sig. |
|---|---|---|---|---|---|
| Between Groups | 4868.488 | 2 | 2434.244 | 3.999 | 0.022 |
| Within Groups | 49913.206 | 82 | 608.698 | | |
| Total | 54781.694 | 84 | | | |

**Multiple Comparisons**

Dependent Variable:AccuracyS4

| | (I) Group | (J) Group | Mean Difference (I-J) | Std. Error | Sig. | 95% Confidence Interval | |
|---|---|---|---|---|---|---|---|
| | | | | | | Lower Bound | Upper Bound |
| Scheffe | 60 | 72 | -13.02941 | 5.98379 | 0.1 | -27.9479 | 1.8891 |
| | | 100 | -18.58824* | 7.32862 | 0.045 | -36.8596 | -0.3169 |
| | 72 | 60 | 13.02941 | 5.98379 | 0.1 | -1.8891 | 27.9479 |
| | | 100 | -5.55882 | 7.32862 | 0.751 | -23.8301 | 12.7125 |
| | 100 | 60 | 18.58824* | 7.32862 | 0.045 | 0.3169 | 36.8596 |
| | | 72 | 5.55882 | 7.32862 | 0.751 | -12.7125 | 23.8301 |

*. The mean difference is significant at the 0.05 level.

**ANOVA**

AccuracyS5

| | Sum of Squares | df | Mean Square | F | Sig. |
|---|---|---|---|---|---|
| Between Groups | 4154.747 | 2 | 2077.374 | 2.418 | 0.095 |
| Within Groups | 70446.265 | 82 | 859.101 | | |
| Total | 74601.012 | 84 | | | |

**ANOVA**

AccuracyS6

| | Sum of Squares | df | Mean Square | F | Sig. |
|---|---|---|---|---|---|
| Between Groups | 4947.688 | 2 | 2473.844 | 3.467 | 0.036 |
| Within Groups | 58502.265 | 82 | 713.442 | | |
| Total | 63449.953 | 84 | | | |

**Multiple Comparisons**

Dependent Variable:AccuracyS6

| | (I) Group | (J) Group | Mean Difference (I-J) | Std. Error | Sig. | 95% Confidence Interval | |
|---|---|---|---|---|---|---|---|
| | | | | | | Lower Bound | Upper Bound |
| Scheffe | 60 | 72 | -2.5 | 6.478 | 0.928 | -18.65 | 13.65 |
| | | 100 | -20.118* | 7.934 | 0.045 | -39.9 | -0.34 |
| | 72 | 60 | 2.5 | 6.478 | 0.928 | -13.65 | 18.65 |
| | | 100 | -17.618 | 7.934 | 0.091 | -37.4 | 2.16 |
| | 100 | 60 | 20.118* | 7.934 | 0.045 | 0.34 | 39.9 |
| | | 72 | 17.618 | 7.934 | 0.091 | -2.16 | 37.4 |

*. The mean difference is significant at the 0.05 level.

## Male Subjects:

**ANOVA**

AccuracyS1

| | Sum of Squares | df | Mean Square | F | Sig. |
|---|---|---|---|---|---|
| Between Groups | 573.518 | 2 | 286.759 | 0.308 | 0.736 |
| Within Groups | 76326.059 | 82 | 930.806 | | |
| Total | 76899.576 | 84 | | | |

**ANOVA**

AccuracyS2

| | Sum of Squares | df | Mean Square | F | Sig. |
|---|---|---|---|---|---|
| Between Groups | 3278.882 | 2 | 1639.441 | 2.018 | 0.139 |
| Within Groups | 66614.412 | 82 | 812.371 | | |
| Total | 69893.294 | 84 | | | |

**ANOVA**

AccuracyS3

| | Sum of Squares | df | Mean Square | F | Sig. |
|---|---|---|---|---|---|
| Between Groups | 13294.451 | 2 | 6647.226 | 2.159 | 0.122 |
| Within Groups | 252507.6 | 82 | 3079.361 | | |
| Total | 265802.05 | 84 | | | |

## Duration

## Female subjects:

**ANOVA**

DurationS4

| | Sum of Squares | df | Mean Square | F | Sig. |
|---|---|---|---|---|---|
| Between Groups | 7512.222 | 2 | 3756.111 | 4.969 | 0.009 |
| Within Groups | 65766.667 | 87 | 755.939 | | |
| Total | 73278.889 | 89 | | | |

**Multiple Comparisons**

Dependent Variable:DurationS4

| | (I) Group | (J) Group | Mean Difference (I-J) | Std. Error | Sig. | 95% Confidence Interval | |
|---|---|---|---|---|---|---|---|
| | | | | | | Lower Bound | Upper Bound |
| Scheffe | 60 | 72 | -11.11111 | 6.48048 | 0.236 | -27.2507 | 5.0285 |
| | | 100 | 13.61111 | 7.93693 | 0.235 | -6.1558 | 33.378 |
| | 72 | 60 | 11.11111 | 6.48048 | 0.236 | -5.0285 | 27.2507 |
| | | 100 | 24.72222* | 7.93693 | 0.01 | 4.9553 | 44.4892 |
| | 100 | 60 | -13.61111 | 7.93693 | 0.235 | -33.378 | 6.1558 |
| | | 72 | -24.72222* | 7.93693 | 0.01 | -44.4892 | -4.9553 |

*. The mean difference is significant at the 0.05 level.

**ANOVA**

DurationS5

| | Sum of Squares | df | Mean Square | F | Sig. |
|---|---|---|---|---|---|
| Between Groups | 870.556 | 2 | 435.278 | 0.224 | 0.8 |
| Within Groups | 169254.17 | 87 | 1945.45 | | |
| Total | 170124.72 | 89 | | | |

**ANOVA**

DurationS6

| | Sum of Squares | df | Mean Square | F | Sig. |
|---|---|---|---|---|---|
| Between Groups | 1052.5 | 2 | 526.25 | 0.295 | 0.745 |
| Within Groups | 155138.89 | 87 | 1783.206 | | |
| Total | 156191.39 | 89 | | | |

**Male Subjects:**

**ANOVA**

DurationS1

| | Sum of Squares | df | Mean Square | F | Sig. |
|---|---|---|---|---|---|
| Between Groups | 3863.611 | 2 | 1931.806 | 1.187 | 0.31 |
| Within Groups | 141631.94 | 87 | 1627.953 | | |
| Total | 145495.56 | 89 | | | |

**ANOVA**

DurationS2

| | Sum of Squares | df | Mean Square | F | Sig. |
|---|---|---|---|---|---|
| Between Groups | 3063.889 | 2 | 1531.944 | 0.481 | 0.62 |
| Within Groups | 277375 | 87 | 3188.218 | | |
| Total | 280438.89 | 89 | | | |

**ANOVA**

DurationS3

| | Sum of Squares | df | Mean Square | F | Sig. |
|---|---|---|---|---|---|
| Between Groups | 17943.333 | 2 | 8971.667 | 6.12 | 0.003 |
| Within Groups | 127538.89 | 87 | 1465.964 | | |
| Total | 145482.22 | 89 | | | |

**Multiple Comparisons**

Dependent Variable:DurationS3

| | (I) Group | (J) Group | Mean Difference (I-J) | Std. Error | Sig. | 95% Confidence Interval | |
|---|---|---|---|---|---|---|---|
| | | | | | | Lower Bound | Upper Bound |
| Scheffe | 60 | 72 | 14.444444 | 9.025 | 0.283 | -8.03119 | 36.92008 |
| | | 100 | 38.611111* | 11.053 | 0.003 | 11.08419 | 66.13803 |
| | 72 | 60 | -14.44444 | 9.025 | 0.283 | -36.92008 | 8.03119 |
| | | 100 | 24.166667 | 11.053 | 0.098 | -3.36026 | 51.69359 |
| | 100 | 60 | -38.611111* | 11.053 | 0.003 | -66.13803 | -11.08419 |
| | | 72 | -24.16667 | 11.053 | 0.098 | -51.69359 | 3.36026 |

*. The mean difference is significant at the 0.05 level.
