# Supplementary material for: Unraveling mysteries of personal performance style; biomechanics of left-hand position changes (shifting) in violin performance
Source: PeerJ. 2015 Oct 1;3:e1299. doi: 10.7717/peerj.1299 (PMC4636401; doi:10.7717/peerj.1299)
Supplement: Supplemental Information 2 [file peerj-03-1299-s003.pdf]

Assessments of expert adjudicators

Subject	Tempo		poor	fair	good	very good	excellent
subject 1	60	evaluate the overall execution of the excerpt				**	*
		evaluate the overall execution of te shifts			*		**
		evaluate the shifting intonation				**	*
		evaluate the timing steadiness of the playing			*	**	
	72	evaluate the overall execution of the excerpt				*	**
		evaluate the overall execution of te shifts			*	**	
		evaluate the shifting intonation				***	
		evaluate the timing steadiness of the playing				***	
	100	evaluate the overall execution of the excerpt				*	**
		evaluate the overall execution of te shifts				*	**
		evaluate the shifting intonation			*	**	
		evaluate the timing steadiness of the playing				**	*
subject 2	60	evaluate the overall execution of the excerpt			*	**	
		evaluate the overall execution of te shifts		*		**	
		evaluate the shifting intonation			***		
		evaluate the timing steadiness of the playing				***	
	72	evaluate the overall execution of the excerpt			*	*	*
		evaluate the overall execution of te shifts			*	**	
		evaluate the shifting intonation			***		
		evaluate the timing steadiness of the playing			*	**	
	100	evaluate the overall execution of the excerpt			*	*	*
		evaluate the overall execution of te shifts		*		**	
		evaluate the shifting intonation			***		
		evaluate the timing steadiness of the playing			*		**
subject 3	60	evaluate the overall execution of the excerpt			***		
		evaluate the overall execution of te shifts			***		
		evaluate the shifting intonation	*	**			
		evaluate the timing steadiness of the playing				***	
	72	evaluate the overall execution of the excerpt	*	**			
		evaluate the overall execution of te shifts		**	*		
		evaluate the shifting intonation		***			
		evaluate the timing steadiness of the playing		*	**		
	100	evaluate the overall execution of the excerpt	**	*			
		evaluate the overall execution of te shifts	*	**			
		evaluate the shifting intonation	**	*			
		evaluate the timing steadiness of the playing		*	**		
subject 4	60	evaluate the overall execution of the excerpt		***			
		evaluate the overall execution of te shifts			***		
		evaluate the shifting intonation	**	*			
		evaluate the timing steadiness of the playing			***		
	72	evaluate the overall execution of the excerpt		**	*		

		evaluate the overall execution of te shifts		*	**		
		evaluate the shifting intonation	*	**			
		evaluate the timing steadiness of the playing			**	*	
	100	evaluate the overall execution of the excerpt	*	*	*		
		evaluate the overall execution of te shifts		*	**		
		evaluate the shifting intonation	*	**			
		evaluate the timing steadiness of the playing			*	**	
subject 5	60	evaluate the overall execution of the excerpt			***		
		evaluate the overall execution of te shifts			**	*	
		evaluate the shifting intonation		***			
		evaluate the timing steadiness of the playing			**	*	
	72	evaluate the overall execution of the excerpt		*	**		
		evaluate the overall execution of te shifts		**	*		
		evaluate the shifting intonation		***			
		evaluate the timing steadiness of the playing		*	**		
	100	evaluate the overall execution of the excerpt		**	*		
		evaluate the overall execution of te shifts		***			
		evaluate the shifting intonation		***			
		evaluate the timing steadiness of the playing			***		
subject 6	60	evaluate the overall execution of the excerpt			*		**
		evaluate the overall execution of te shifts			*		**
		evaluate the shifting intonation		*			**
		evaluate the timing steadiness of the playing				*	**
	72	evaluate the overall execution of the excerpt		*			**
		evaluate the overall execution of te shifts			*		**
		evaluate the shifting intonation		*			**
		evaluate the timing steadiness of the playing				***	
	100	evaluate the overall execution of the excerpt		*			**
		evaluate the overall execution of te shifts			*		**
		evaluate the shifting intonation	*			**	
		evaluate the timing steadiness of the playing			*	*	*
